# SerpinB7 deficiency contributes to development of psoriasis via calcium-mediated keratinocyte differentiation dysfunction

**DOI:** 10.1038/s41419-022-05045-8

**Published:** 2022-07-21

**Authors:** Huaping Zheng, Linna Gu, Fulei Zhao, Chen Zhang, Zhen Wang, Hong Zhou, Zhonglan Hu, Xiaoqiong Wei, Xiao Liu, Feng Luo, Fanlian Zeng, Qixiang Zhao, Yan Hao, Yawen Hu, Xiaoyan Wang, Jing Hu, Jiadong Yu, Wenling Wu, Yifan Zhou, Pei Zhou, Chengcheng Yue, Nongyu Huang, Kaijun Cui, Wei Li, Jiong Li

**Affiliations:** 1 State Key Laboratory of Biotherapy and Cancer Center, West China Hospital, West China Medical School, Sichuan University and Collaborative Innovation Center for Biotherapy, Chengdu, China; 2 Institutes for Systems Genetics, Frontiers Science Center for Disease-related Molecular Network, National Clinical Research Center for Geriatrics, West China Hospital, Sichuan University, Chengdu, Sichuan China; 3 State Key Laboratory of Oral Diseases, National Clinical Research Center for Oral Diseases, West China School of Stomatology, Sichuan University, Chengdu, 610041 China; 4 Department of Cardiovascular Medicine, West China Hospital, Sichuan University, Chengdu, China; 5 Department of Dermatovenereology, West China Hospital, Sichuan University, Chengdu, China

**Keywords:** Cell growth, Psoriasis

## Abstract

Defective execution of proteases and protease inhibitors that mediate abnormal signaling cascades is emerging as a key contributor to skin diseases, such as psoriasis. SerpinB7 is identified as a skin-specific endogenous protease inhibitor, but the role and underlying mechanism in psoriasis are poorly understood. Here we found that SerpinB7 is highly expressed in psoriatic keratinocytes of patients and imiquimod-induced psoriatic lesions in mice. SerpinB7^-/-^ mice showed abnormal epidermal barrier integrity and skin architecture in homeostasis, and aggravated psoriatic lesion with inhibiting terminal differentiation and increasing inflammatory cells infiltration compared to SerpinB7^+/+^ mice after Imiquimod treatment. Mechanistically, SerpinB7 deficiency results in excessive proliferation and impaired differentiation, as well as increased chemokines and antimicrobial peptide expression in normal human epidermal keratinocyte and mouse primary keratinocyte. Transcriptomics and proteomics results showed that the SeprinB7 deficiency affected keratinocyte differentiation and proinflammatory cytokines, possibly by affecting the calcium ion channel-related proteins. Notably, we demonstrated that SerpinB7 deficiency prevented the increase in intracellular Ca^2+^ influx, which was partly eliminated by the intracellular Ca^2+^ chelator BAPTA-AM. Our findings first described the critical role of SerpinB7 in the regulation of keratinocyte differentiation and psoriatic microenvironment mediated via keratinocytes' intracellular calcium flux, proposing a new candidate for therapeutic targets in psoriasis.

## Introduction

Psoriasis, which affects approximately 2% of the global population [1], is a common chronic inflammatory skin disease triggered by a dysregulated immune response that is characterized by excessive hyperproliferation and aberrant differentiation of keratinocytes [1]. A controlled and coordinated balance between immune defense and keratinocyte proliferation and differentiation is essential for the homeostatic of the skin microenvironment [2]. Keratinocytes play a vital role in maintaining an intact epidermal barrier to prevent water loss and against environmental triggers. In the immunopathogenesis of psoriasis, keratinocytes respond to triggers such as injury, infection, or cytokine stimulation, and produce chemokines (CXCL1, CXCLl2, CCL20), antimicrobial peptides (S100A7, S100A8, S100A9, S100A12), DEFB4a/DEFB2, CAMP/LL37, and other inflammatory factors attract and activate pathogenic T cells (T17, Th1 and Th22) and neutrophils [3]. The cytokines released by these immune cells further stimulate keratinocytes, thereby amplifying the immune circuit [4]. The crosstalk between keratinocytes and immunocytes induces and regulates psoriasis. Therefore, psoriatic keratinocytes play a pivotal role in psoriatic skin inflammation.

Proteases and their inhibitors are throughout the epidermis produced by keratinocytes [5]. The degree of cooperation among epidermal proteases is crucial, and the various function-specific cascades may represent different branches of a larger, integrated proteolytic network, as well as protein degradation and processing enzymes; however, they also function as key signaling molecules in an expanding set of cellular pathways [6]. Defective regulation and execution of protease-mediated processes are emerging as key contributors to diverse human skin pathologies, affecting a broad spectrum of biological processes, including keratinocyte proliferation, differentiation, cornification, desquamation, and immune system regulation [5]. ADAM17 deficiency causes inflammatory skin and bowel syndrome, characterized by chronic inflammation, erythema, and scaling, which is presumably linked to defective EGF receptor ligand shedding [7, 8]. The absence of LEKTI propagates an uncontrolled KLK cascade, causing stratum corneum detachment, lipid defects, inflammation, and severe allergic manifestation [9, 10]. The prevalence of multiple diseases attributable to aberrant proteolytic activity, powerfully demonstrates that proteases and their inhibitors are crucial for epidermal homeostasis.

Serine protease inhibitors (Serpins) form a superfamily of proteins that share a conserved tertiary structure [11], and are involved in diverse physiological processes, including cell growth, fibrinolysis and inflammation to immunity [12]. A previous study showed that SerpinB7 is upregulated in diabetic nephropathy and induces progressive mesangial expansion [13]. A recent finding showed that loss-of-function mutation of c.796C>T (p. Arg266*) in *SerpinB7* causes autosomal recessive Nagashima-type palmoplantar keratosis (NPPK), resulting in loss of integrity of the stratum corneum of the epidermis, enhancing water permeation [14], suggesting that SerpinB7 is a crucial element in skin homeostasis. In our previous study, we showed that SerpinB7-specific high expression in skin tissue was identified as a skin-specific psoriatic pathogenic molecule [15]. However, the role of SerpinB7 in skin homeostasis and psoriasis pathophysiology remains unknown.

In this study, we investigated the correlation between the expression of SerpinB7 and psoriasis, and determined whether it has potential functional relevance in psoriasis. We identified SerpinB7 expression in keratinocytes as an important player in the skin homeostasis and amplification of psoriasiform inflammation and revealed that SerpinB7 induces keratinocyte differentiation and inhibits the production of various chemokines and antimicrobial peptides in a calcium-dependent manner. Our findings revealed a vital component that appears to be inherent in keratinocyte differentiation and is of potential importance to the pathogenesis of psoriasis.

## Results

### SerpinB7 is abundantly expressed in epidermal keratinocytes of psoriasis patients and imiquimod-induced psoriatic skin lesions

**Fig. 1:**
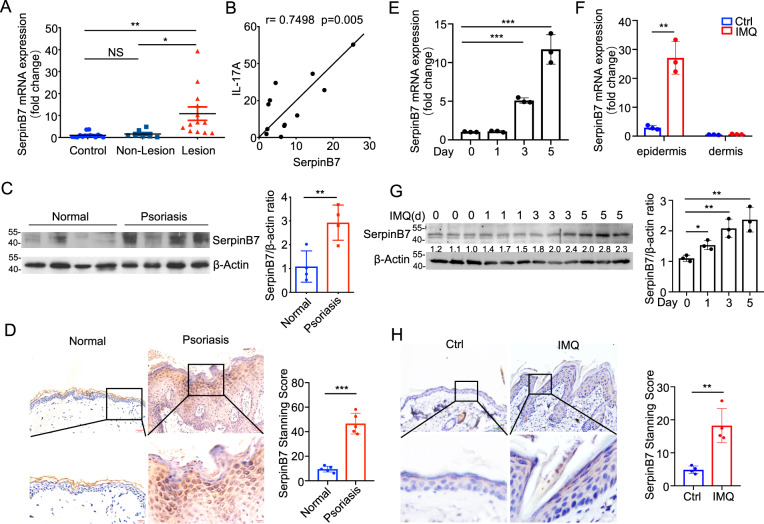
SerpinB7 is abundantly expressed in epidermal keratinocytes. **A** Expression of *SerpinB7* mRNA in normal (*n* = 15), non-lesional (*n* = 7), and lesional (*n* = 12) human psoriatic skin. **B** Correlation plot between *SerpinB7* and *IL-17a* in human psoriatic lesion skin. **C** Immunoblot analysis of protein level of SerpinB7 in skin extracts from normal and psoriasis patients. β-actin was used as an internal control. **D** Immunohistochemistry staining of SerpinB7 in the skin from healthy and psoriasis patients, scale bar: overviews (100 μm) and insets (20 μm) (left), quantitative analysis of four fields per mouse (right), *n* = 5. **E** Expression of SerpinB7 mRNA of back skin from treated IMQ or control cream mice. **F** The *SerpinB7* mRNA levels was measured by RT-PCR from dissected mice's epidermis and dermis. **G** Immunoblot analysis of protein level of SerpinB7 in skin extracts from normal and psoriasis patients. β-actin was used as an internal control. **H** Immunohistochemistry staining the skin from treated IMQ or control cream mice. scale bar: overviews (100 μm) and insets (20 μm) (left), quantitative analysis of four fields per mouse (right), *n* = 4. Mean ± SD. *P < 0.05; **P < 0.01; ***P < 0.001. Two-tailed Student’s *t*-test (**C**–**F** and **H**), one-way ANOVA (**A** and **G**). All the data are representative of three independent experiments.

Psoriasis is a skin disease characterized by excessive epithelial cell proliferation and enhanced antimicrobial defense [16], Since our previous study suggested that SerpinB7 is a skin-specific molecule associated with psoriasis [15], we first examined the expression of SerpinB7 in skin biopsies from 13 patients with psoriasis. Compared to healthy people and non-lesion skin of psoriasis patients, patients with psoriasis had significantly increased *SerpinB7* mRNA in lesional skin (Fig. 1A), and a positive correlation between the expression of psoriasis severity marker IL-17 and SerpinB7 (Fig. 1B). The GEO profile (GDS4602, GDS4600, and GDS5420) also showed that lesional skin was strongly enhanced compared to normal and non-lesional skin (Supplementary Fig. 1A–C), further downregulated following treatment with brodalumab, an IL-17RA mAb (Supplementary Fig. 1C). Consistently, immunoblot and immunofluorescent staining showed that the SerpinB7 was abundantly increased in lesion skin of psoriasis patients compared to healthy control, specifically localized to epidermal keratinocytes (Fig. 1C, D). To further confirm that SerpinB7 is related to the psoriatic phenotype, we evaluated an imiquimod (IMQ)-induced psoriasis-like mouse model [17] and found that both mRNA and protein levels of SerpinB7 were increased in an IMQ-induced psoriasis-like mouse model with a time-dependent increase in the psoriatic epidermis (Fig. 1E–G). Moreover, IL-17A, IL-22, oncostatin M, TNF-α, and IL-1α (M5) synergistically stimulated keratinocytes as an in vitro psoriatic model, were gradually upregulated from 0 to 48 h (Supplementary Fig. 2).

### SerpinB7 deficiency results in postnatal epidermal barrier defects

**Fig. 2:**
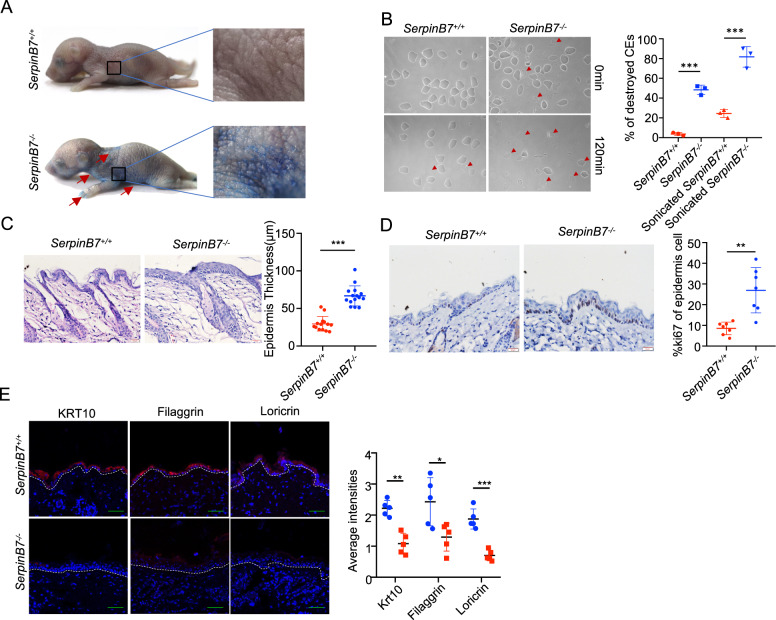
SerpinB7 deficiency results in postnatal epidermal barrier defects and worsens psoriasis symptoms. **A** Toluidine blue dye-penetration assay was performed with newborn SerpinB7^+/+^ and Serpinb7^-/-^ mice indicating outside-in barrier defects. **B** SerpinB7^+/+^ and Serpinb7^-/-^ mice CEs were isolated and treated with ultrasound for 120 min. CE destruction is indicated by the triangle. **C** Representative H&E staining section (20×) from the skin tissues of SerpinB7^+/+^ and Serpinb7^-/-^ mice. Right: statistical analysis of skin epidermal thickness (mean ± SEM). **D** Representative immunohistochemical staining (20×) of Ki67 in the dorsal skin of SerpinB7^+/+^ and Serpinb7^-/-^ mice. Right: quantification of Ki67-positive cells in the epidermis. **E** Representative Immunofluorescent staining of Krt10, Filaggrin, and Loricrin in the dorsal skin of SerpinB7^+/+^ and Serpinb7^-/-^ mice.

To assess the role of SerpinB7 in the skin, we established a SerpinB7 knockout mouse (SerpinB7^-/-^) using CRISPR/Cas-mediated genome engineering (Supplementary Fig. 3A), and offspring genotypes were verified through PCR amplification using three primer pairs (Supplementary Fig. 3B). We further confirmed the absence of SerpinB7 expression in SerpinB7^-/-^ at the mRNA and protein level (Supplementary Fig. 3B–D). Although SerpinB7^-/-^ mice were born at the expected Mendelian ratio and presented a body size that was comparable to that of control littermates (Supplementary Fig. 3E, F), the skin of newborn SerpinB7^-/-^ mice was characterized by an unusually shiny, translucent and more finely wrinkled morphology compared with SerpinB7^+/+^ mice. Epidermal barrier defects lead to water loss and contribute to psoriasis development [18]. Therefore, we used a dye-penetration assay to examine the integrity of the skin barrier. The epidermis of the newborn SerpinB7^+/+^ mice was completely resistant to toluidine blue penetration, whereas SerpinB7^-/-^ mice showed dye permeability in the mechanically stressed areas, including the paw, neck, and subscapular (Fig. 2A). Impaired integrity of cornified envelope (CE) has been linked to skin barrier defects [19]. CE preparations derived from the postnatal biopsies of the SerpinB7^-/-^ mouse dorsal skin showed structural abnormalities and fragility, and the percentage of intact CE from the SerpinB7^-/-^ mouse epidermis decreased drastically after 120 min of ultrasound treatment (Fig. 2B). Histological analysis revealed epidermal hyperplasia in 8-week-old Serpinb7^-/-^ mice (Fig. 2C), associated with enhanced proliferation of keratinocytes, as evidenced by a three-fold increase in the number of Ki67-positive cells in the stratum basale (Fig. 2D). To explore the basis for skin barrier defects observed in SerpinB7^-/-^ mice, we determined the alterations in the expression of keratinocyte differentiation markers keratin-10 (KRT10), filaggrin (FLG), and loricrin (LOR) using immunofluorescence, showed nearly disappeared within the epidermis of SerpinB7^-/-^ mice (Fig. 2E). Thus, while SerpinB7 deficiency causes defective epidermis barrier function, but not impaired pathological feature thereafter.

### Deficiency of SerpinB7 aggravates skin inflammation and epidermal hyperplasia in IMQ-induced psoriasis-like model

**Fig. 3:**
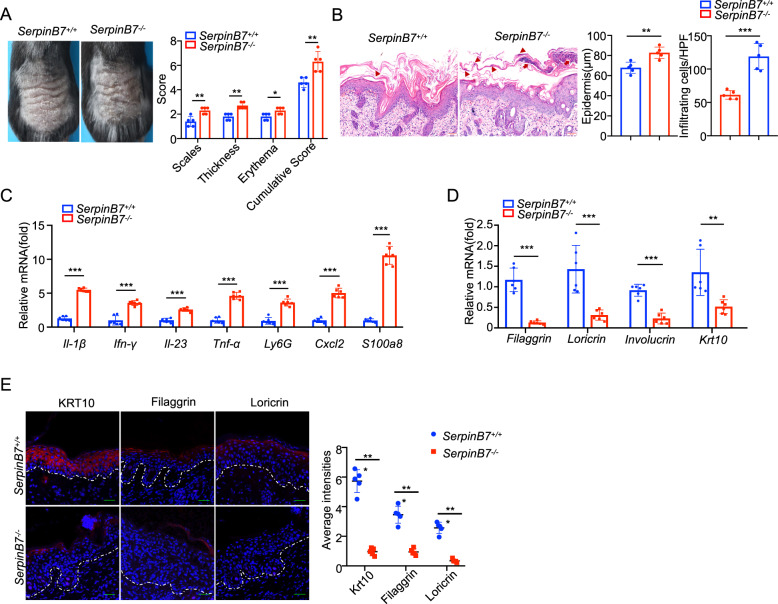
Ablation of SerpinB7 leads to exacerbation of IMQ-induced Psoriasis model. **A** SerpinB7^+/+^ and SerpinB7^-/-^ mice were treated with IMQ for 5 days, *n* = 5/group. Representative images of the dorsal skin from mice (left) and mice PASI scores were depicted (right). **B** Representative H&E staining section from the skin tissues of SerpinB7^+/+^ (*n* = 5) and SerpinB7^-/-^ (*n* = 5) mice. Parakeratosis is indicated by the triangle, Munro’s microabscess is indicated by the arrows. Right: statistical analysis of skin epidermal thickness and inflammatory cell infiltration. **C** The RNA levels of keratinocytes differentiation-related genes were assessed by qPCR. **D** The RNA levels of chemokines and antimicrobial peptides were assessed by qPCR. **E** Immunofluorescent staining of Krt10, Filaggrin, and Loricrin in the dorsal skin of SerpinB7^+/+^ (*n* = 5) and Serpinb7^-/-^ (*n* = 5) mice. Right: statistical analysis of staining intensity in the epidermis. Mean ± SD. *P < 0.05; **P < 0.01; ***P < 0.001. Two-tailed Student’s *t*-test (**A**–**E**). All the data are representative of three independent experiments.

To explore the potential role of SerpinB7 in psoriasis, we subjected SerpinB7^-/-^ mice to IMQ-induced psoriatic model, which exhibited a significant increase in the mouse Psoriasis Area and Severity Index (PASI), evaluates erythema, scales, and thickness [20] compared to those of SerpinB7^+/+^ mice (Fig. 3A). The histology of SerpinB7^-/-^ IMQ-induced psoriatic mouse skin displayed significant elevated inflammatory cell infiltration and epidermal thickness with increased development of pustules compared to SerpinB7^+/+^ mice (Fig. 3B). These results suggest that SerpinB7 may play a crucial role in psoriasis. Moreover, SerpinB7 deficiency affected IMQ-induced skin inflammation, and showed increased expression of chemokines (*TNF-α, IL-1b, IL-23*, and *IFN-γ*), neutrophil chemotactic factors *CXCL2*, neutrophil marker *Ly6G* and antimicrobial peptide *S100A8* in skin lesions compared with SerpinB7^+/+^ (Fig. 3C). In addition, we observed a significant decrease in the expression of the skin differentiation marker genes *KRT10*, *FLG*, and *LOR* in IMQ-treated SerpinB7^-/-^ mice (Fig. 3D). Immunofluorescence analysis confirmed altered epidermal differentiation in IMQ-treated SerpinB7^-/-^ mice, with decreased expression of KRT10, FLG, and LOR, KRT10 expression was absent in the stratum of spinous compared to SerpinB7^+/+^ mice (Fig. 3E), suggesting exacerbated dysregulation of keratinocyte differentiation. Furthermore, an rhIL-23-induced psoriatic inflammatory model showed that SerpinB7 deficiency elevated epidermal thickness (Supplementary Fig. 4A), increased the expression of chemokines and antimicrobial peptides (Supplementary Fig. 4B), and decreased keratinocyte differentiation markers (Supplementary Fig. 4C). Therefore, our data imply that the SerpinB7 deficiency exacerbates psoriasis, leading to increased levels of inflammatory factors and dysregulated epidermal differentiation.

To evaluate the function of SerpinB7 in psoriasiform keratinocytes, we established lentiviral-transfected SerpinB7 shRNA and SerpinB7-overexpressing HaCaT cells, which are immortalized human keratinocytes. The efficiency of the knockdown and overexpression systems was determined using RT-PCR (Supplementary Fig. 5A, B). SerpinB7 knockdown in HaCaT cells drastically increased the expression of psoriasis-related chemokines (*CCL20, CCL27, CXCL1, CXCL2*) and antimicrobial peptides *LL37* and *S100A8* in the M5-stimulated in vitro psoriatic model (Supplementary Fig. 5C). Conversely, ectopic SerpinB7 expression significantly decreased the mRNA levels of chemokines (*CCL20, CCL27, CXCL1, CXCL2*, and *CXCL8*) and antimicrobial peptides (*BD2, LL37, S100A7-A9*, and *S100A12*) in M5-stimulated HaCaT cells (Supplementary Fig. 5D).

### SerpinB7 deficiency promotes proliferation and suppresses differentiation in keratinocytes

Psoriasis is characterized by typical histopathological features including abnormally increased proliferation and hampered differentiation of keratinocytes leading to epidermal acanthosis and parakeratosis [4]. The normal epidermis has a Ca^2+^ gradient that is disturbed in psoriatic plaques, with drastically reduced Ca^2+^ influx in psoriatic keratinocytes, favoring abnormal keratinocyte proliferation and differentiation [21]. First, we analyzed the relationship between SerpinB7 expression and keratinocyte-related genes by analyzing the RNA-seq data of 607 skin tissue samples obtained from the GTEx database (Supplementary Fig. 6A). We sorted the RNA profiles based on the *SerpinB7* mRNA expression levels and the 30 tissues exhibiting high and low expression were divided into high SerpinB7 and low SerpinB7 groups, respectively (Supplementary Fig. 6B). We then compared the expression of genes involved in the gene ontology (GO) terms by performing hierarchical clustering, which showed a strong correlation between *keratinocyte differentiation* *genes* and SerpinB7 expression (Supplementary Fig. 6C). Gene set enrichment analysis also indicated a significant correlation between the SerpinB7 and keratinocyte differentiation, as well as skin development (Supplementary Fig. 6D). We then evaluated SerpinB7 expression during keratinocyte differentiation by adopting a well-established 1.6 mM calcium-induced differentiation model [22]. RT-PCR and immunoblot results showed the expression of SerpinB7 and keratinocyte differentiate genes *LOR*, *FLG*, and *KRT10* were significantly increased in differentiated keratinocytes (Supplementary Fig. 6E–G) and decreased with the expression of keratinocyte development gene *KRT5* (Supplementary Fig. 6G).

**Fig. 4:**
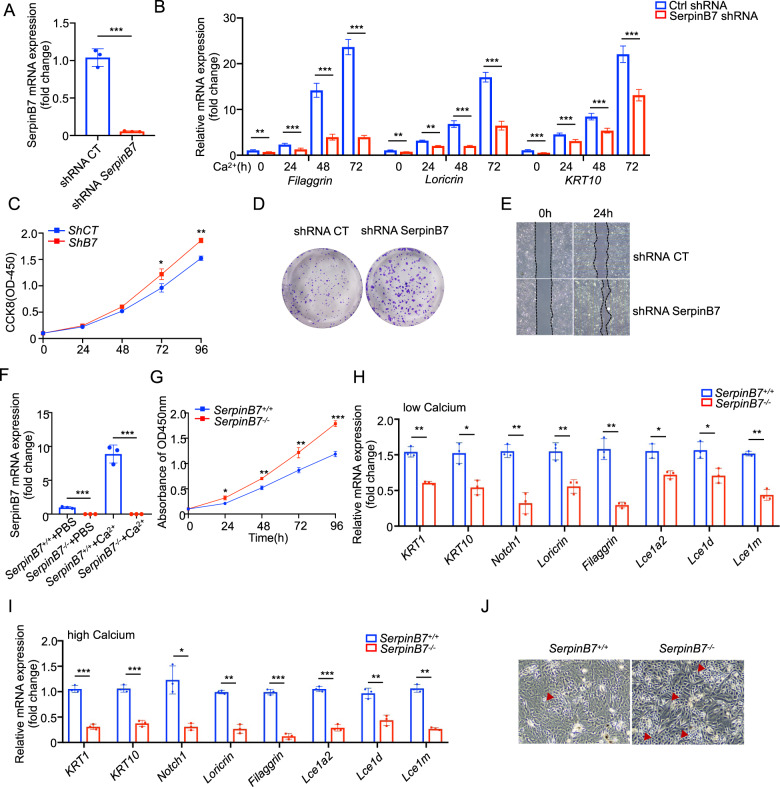
SerpinB7 deficiency promotes proliferation and suppresses differentiation in keratinocytes. **A** NHEK cells were transfected with shCT and sh-SerpinB7. The mRNA level of *SerpinB7* was measured by RT-PCR. **B** CaCl_2_-induced *Filaggrin, Loricrin, and KRT10* expression in shCT and sh-SerpinB7 NHEKs at 0–72 h was measured by RT-PCR. **C**, **D** The proliferation of sh-SerpinB7 keratinocytes was evaluated with a CCK8 (Cell Counting Kit 8) assay and colony formation assay. **E** Identical fields scratch of undifferentiated sh-SerpinB7 and control NHEKs at 0 and 24 h. The dotted line represents the area of the scratch. **F** The mRNA level of SerpinB7 in Serpinb7^-/-^ and Serpinb7^+/+^mice primary keratinocytes (MKs) was measured by RT-PCR. **G** The proliferation of SerpinB7^+/+^ and Serpinb7^-/-^ MKs was evaluated with a CCK8 assay. **H**, **I** SerpinB7^+/+^ and Serpinb7^-/-^ MKs homeostasis and treated for 24 h with 1.6 mM CaCl^2+^ were analyzed by RT-PCR for the expression of early (*Krt1, Krt10*) and late (*Notch1, Loricrin, Filaggrin, Lce1a2, Lce1d, Lce1m*) differentiation genes. **J** Morphology of SerpinB7^+/+^ and Serpinb7^-/-^ MKs in response to Ca^2+^ induced differentiation, red arrow indicated typical undifferentiated keratinocyte state. Mean ± SD. *P < 0.05; **P < 0.01; ***P < 0.001. Two-tailed Student’s *t*-test (**A**, **H**, **I**), one-way ANOVA (**B**, **C**, **F**, **G**). All the data are representative of three independent experiments.

To examine whether SerpinB7 is essential for suppressing the growth and promoting the differentiation of keratinocytes, we conducted loss-of-function studies. normal human epidermal keratinocyte (NHEK) cells were infected with lentivirus carrying either *SerpinB7* shRNA or scrambled shRNA as a control, and efficient knockdown of SerpinB7 was validated using RT-PCR (Fig. 4A). We have observed that the knockdown of SerpinB7 in undifferentiated and differentiated keratinocytes inhibited the expression of *KRT10*, *FLG*, and *LOR* expressions (Fig. 4B). This suggests that SerpinB7 deficiency may inhibit keratinocyte differentiation and promote keratinocyte proliferation. In comparison to the scramble control, SerpinB7 knockdown promotes the growth of NHEK in morphology and CCK8 results (Fig. 4C), a nearly three-fold increase in colony-forming efficiency was observed (Fig. 4D), and the migration ability of keratinocytes was increased (Fig. 4E). Furthermore, we isolated SerpinB7^-/-^ primary mice keratinocytes (MKs) and showed absence in basal and differentiated conditions (Fig. 4F). Under conditions that maintain MKs in an undifferentiated state, CCK8 results determined that SerpinB7 deficiency promotes MK's proliferation (Fig. 4G). In contrast to basal genes whose expression was not significantly affected, most of the differentiation genes examined were significantly downregulated by SerpinB7^-/-^ MKs (Fig. 4H). After calcium-induced differentiation in vitro, SerpinB7^-/-^ MKs showed significantly upregulated basal genes and downregulated differentiation genes (Fig. 4I), and displayed a smaller and denser, cobblestone-like undifferentiated phenotype (Fig. 4J). Together, these findings indicate that SerpinB7 deficiency decreased the propensity of basal cells to undergo differentiation.

### SerpinB7 deficiency affects the expression of genes related to calcium channels in differentiated keratinocytes

**Fig. 5:**
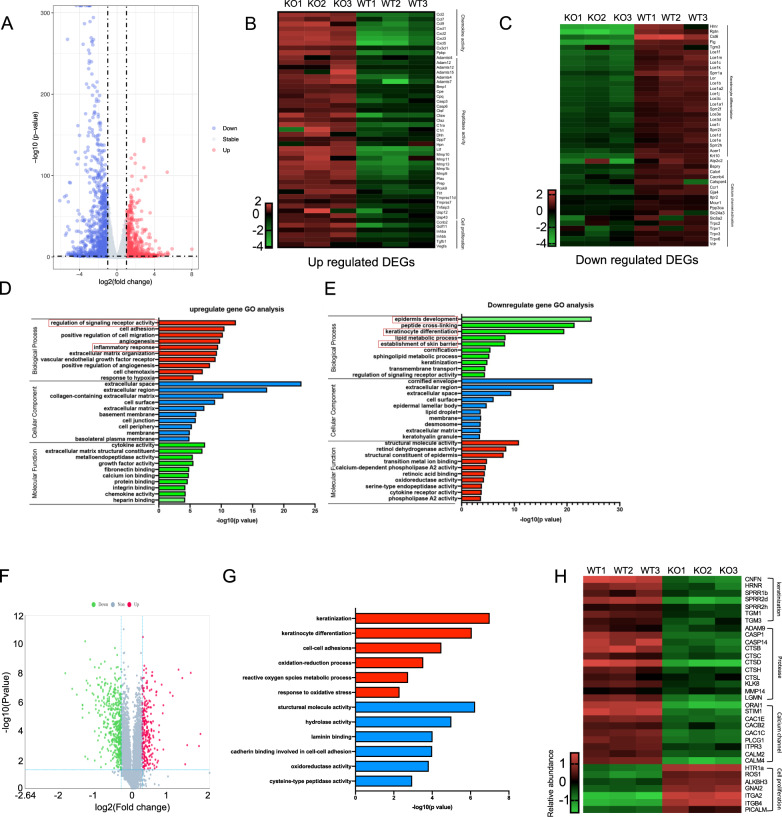
SerpinB7 deficiency affects the expression of genes related to calcium channels in differentiated keratinocytes. **A** Volcano plots plotting the log2 fold-change of transcriptomics in SerpinB7 deficiency vs. control differentiated keratinocyte. Blue color indicates transcripts with fold-change (FC) < 0.67 while red color those with FC > 1.5. **B**, **C** Heatmaps showing the upregulated (left) and downregulated (right) genes in SerpinB7 deficiency differentiated keratinocytes. **D**, **E** The top 10 most relevant biological processes, cellular components, and Molecular function gene ontology (GO) are sorted according to the adjusted *P* value. **F** Volcano plots plotting the log2 fold-change of proteomics in SerpinB7 deficiency vs. control differentiated keratinocyte. Blue color indicates transcripts with FC < 0.83 while red color those with FC > 1.2. **G** The top 5 most relevant biological processes upregulated and downregulated GO are sorted according to the adjusted *P* value. **H** Heatmaps showing the keratinization, protease, calcium channel, and cell proliferation DEGs.

To further investigate the function of SerpinB7 in differentiated keratinocytes, RNA-seq was performed to extensively evaluate the gene expression profile in differentiated lentivirus-transfected NHEK cells with *SerpinB7* shRNA or scrambled shRNA. Among the 17,508 distinct transcripts detected, 582 and 996 mRNA were significantly upregulated and downregulated, respectively, compared to the controls. (Fig. 5A and Supplementary Table 1). A heatmap of 1166 differentially expressed genes (DEGs) shows that gene-level clustering was driven by sample type; the DEG clusters of molecular functions that were upregulated by SerpinB7 deficiency were related to chemokine activity, peptidases activity, and cell proliferation (Fig. 5B). Downregulated DEGs were related to keratinocytes differentiation and calcium channel activity (Fig. 5C). Within the differentiated expression genes using a fold-change threshold of 2 and *P* < 0.01. GO analysis revealed that upregulated genes were clustered in the regulation of signaling receptor activity, cell adhesion, positive regulation of cell migration, and inflammatory response (Fig. 5D). Downregulated genes were mainly clustered in keratinocyte differentiation, peptide cross-linking, and epidermis development (Fig. 5E). In addition, the Kyoto Encyclopedia of Genes and Genomes pathway analysis of these DEGs identified the cytokine–cytokine receptor interactions and calcium signaling pathway (Supplementary Fig. 7A, B). To explore possible interactions in the relationships of the 102 differentially expressed proteins, the STRING online database was utilized to calculate protein–protein interaction scores, downregulated DEGs were more enriched in processes involving keratinocyte differentiation (*CASP14, KRT1, KRT10, HRNR SPRR2H*), upregulated DEG more enriched in processes involving inflammatory (*CCL20, CXCL1, CXCL2, CXCL5, TNF, VEGFA*) (Supplementary Fig. 7C, D). These results suggested that SerpinB7 contributes to the keratinocyte differentiation and inflammatory mediator expression by influencing protease activity and calcium ion channels.

Furthermore, proteomic was performed in differentiated SerpinB7 knockdown NHEK cells. We identified 32,317 peptides and 5857 proteins (peptide false discovery rate ≤0.01) through proteomics (Fig. 5F). GO enrichment analysis of 647 DEGs (fold-change >1.2, *P* value < 0.05) showed that they principally participated in keratinization, keratinocyte differentiation, calcium signaling pathway, oxidation-reduction process in terms of biological process (Fig. 5G) and structural molecular activity, hydrolase activity, oxidoreductase activity in terms of molecular function (Fig. 5G). A complete list of the genes and GO categories is provided in Supplementary Table 2. Heatmap analysis of the differential expression of important representative genes associated with these main pathogenesis-related functions is shown (Fig. 5H). Among the representative genes, SerpinB7 deficiency significantly decreased keratinization, protease, calcium channel-related protein expression, and increased cell proliferation-related protein expression (Fig. 5H).

These results are consistent with those regarding the biological functions of SerpinB7 that may be involved in psoriasis pathology, demonstrating that SerpinB7 is critical for keratinocyte differentiation and keratinocytes differentiation.

### SerpinB7 affected keratinocytes differentiation and inflammation by regulating Ca^2+^ influx

**Fig. 6:**
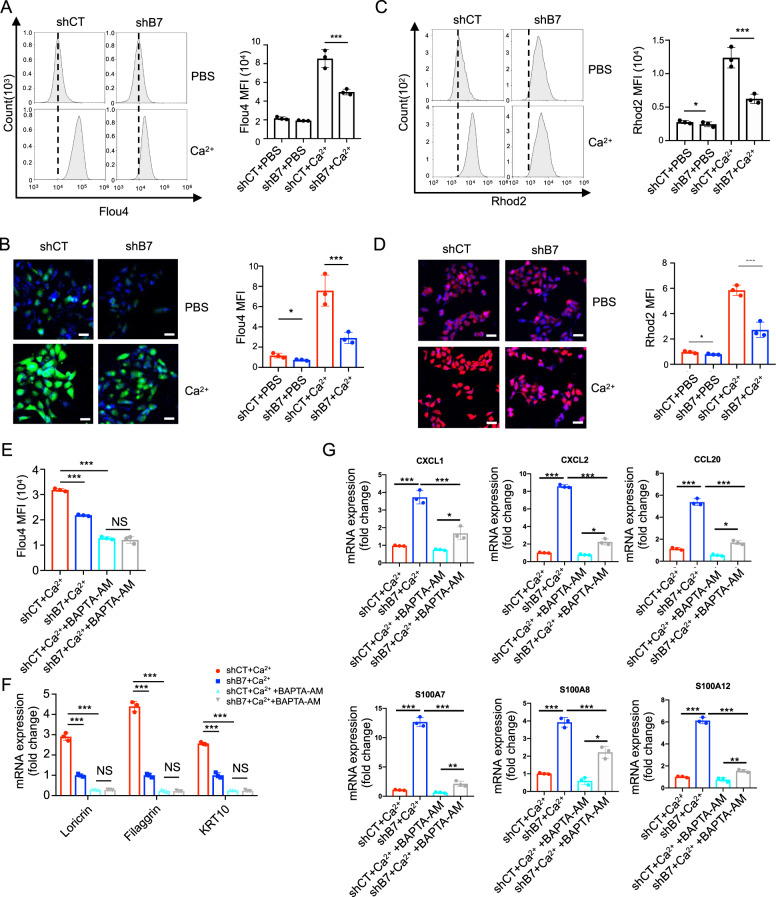
SerpinB7 affected keratinocytes differentiation and inflammation via regulating Ca^2+^ influx. **A**, **B** Ca^2+^ concentration in cytosol ([Ca^2+^]_c_) and **C**, **D** Ca^2+^ concentration in mitochondria ([Ca^2+^]_m_) of shSerpinB7 and shCT NHEKs homeostasis and treated for 24 h with 1.6 mM Ca^2+^ were measured by flow cytometry and fluorescence confocal imaging using Flou4-AM and Rhod2-AM, respectively. **E** [Ca^2+^]_c_ of Ca^2+^-induced shSerpinB7 and shCT NHEKs treated with BAPTA-AM were measured by flow cytometry using Flou4-AM. **F** The mRNA level of keratinocyte differentiation markers in Ca2+-induced shSerpinB7 and shCT NHEKs treated with BAPTA-AM was measured by RT-PCR. **G** The mRNA level of chemokines and microbial peptides in Ca^2+^-induced shSerpinB7 and shCT NHEKs treated with 20 um/mL BAPTA-AM was measured by RT-PCR. Flou4-AM: intracellular calcium indicator, Rhod2-AM mitochondrial Calcium indicator, BAPTA-AM, Mean ± SD. *P < 0.05; **P < 0.01; ***P < 0.001. Two-tailed Student’s *t*-test (**A**–**D**), one-way ANOVA (**E**–**G**). All the data are representative of three independent experiments.

Previous studies have suggested that SerpinB7 deficiency influences calcium channels. An increase in the extracellular Ca^2+^ concentration leads to an increased cytosolic Ca^2+^ and an overload of Ca^2+^ in the mitochondria, depolarizing and consequently inducing keratinocyte differentiation [22]. To assess the consequences of SerpinB7 knockdown affection in differentiated keratinocytes, we monitored Ca^2+^-induced changes in ionized Ca^2+^ concentration in the cytosol ([Ca^2+^]_c_) and mitochondria ([Ca^2+^]_m_), using flow cytometry and fluorescence confocal imaging. Changes in [Ca^2+^]_c_ and [Ca^2+^]_m_ in response to the external application of 1.6 mM Ca^2+^ were measured after 24 h, using Flou4 and Rhod2, respectively (Fig. 6A–D). Compared to the control NHEKs, the [Ca^2+^]_c_ and [Ca^2+^]_m_ detected in SerpinB7 knockdown NHEKs were significantly diminished (Fig. 6A–D). In addition, exogenous rhSerpinB7 and lentivirus-overexpressed SerpinB7 significantly increased [Ca^2+^]_c_ in NHEKs after 1.6 mM Ca^2+^ induced (Supplementary Fig. 8A), along with increased expression of keratinocyte differentiation and calcium channel-related markers (Supplementary Fig. 8B, C). These findings suggest that SerpinB7 deficiency impedes keratinocyte differentiation by attenuating [Ca^2+^]_c_ and [Ca^2+^]_m_.

To further investigate the effect of Ca^2+^ influx on keratinocyte differentiation and inflammatory chemokine expression, BAPTA-AM, an intracellular Ca^2+^ chelator was used to inhibit cytosolic Ca^2+^ concentration [23]. The results showed that BAPTA-AM treatment drastically blocked the cytosolic Ca^2+^ elevation and significantly eliminated the difference between differentiated SerpinB7 knockdown and control NHEKs (Fig. 6E). Furthermore, BAPTA-AM markedly diminished the expression of the differentiation markers FLG, LOR, KRT10 in Ca^2+^-induced keratinocytes, eliminating the differences in differentiation marker expression between SerpinB7 knockdown and control NHEKs (Fig. 6F). In addition, 10 μM BAPTA-AM significantly inhibited Ca^2+^ induced increases in chemokines and antimicrobial peptides, whereas BAPTA-AM attenuated the difference caused by SERPINB7 deficiency (Fig. 6G). These results suggest that the abnormal expression of differentiation markers, chemokines, and antimicrobial peptides in SerpinB7 deficiency differentiated keratinocytes may be due to the decreased [Ca^2+^]_m_.

## Discussion

**Fig. 7:**
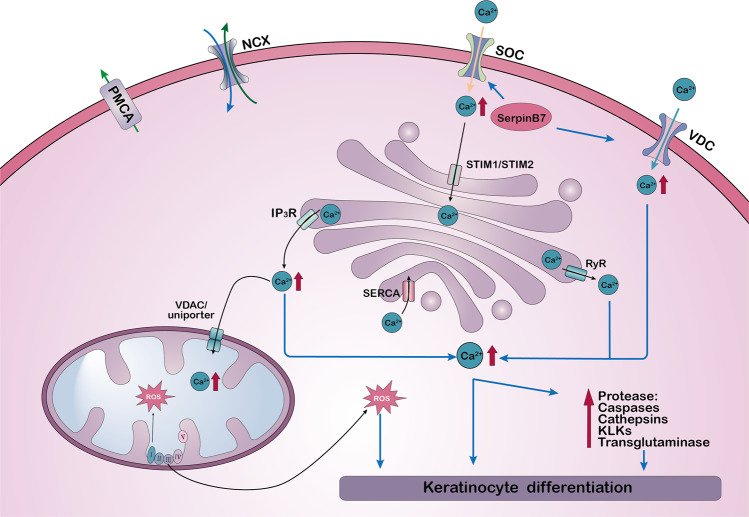
Schematic representation of the function of SerpinB7 in keratinocyte.

In this study, we have characterized SerpinB7 as a new psoriatic candidate gene and found a significant positive correlation between the expression of SerpinB7 and psoriasis severity. A previous study showed that SerpinB7 contributes to extracellular matrix accumulation in diabetic glomeruli and induces progressive mesangial expansion in mice [13, 24]. Although mutations in SerpinB7 cause NPPK, which is an autosomal recessive non-syndromic diffuse palmoplantar keratosis [14], the study on the biological function of SerpinB7 in the skin is poorly understood. Here, we have observed that SerpinB7 deficiency disrupts the skin barrier and exacerbates IMQ-induced psoriatic inflammation. We further demonstrated that SerpinB7 deficiency inhibited keratinocyte differentiation and promoted the expression of inflammatory cytokines and antimicrobial peptides by attenuating [Ca^2+^]_c_ and [Ca^2+^]_m_ concentrations Fig. 7. These findings reveal a novel keratinocyte-specific molecule that is involved in psoriasis pathogenesis and the mechanism through which SerpinB7 affects [Ca^2+^]_c_ and [Ca^2+^]_m_, to regulate keratinocyte differentiation and inflammatory response.

The integrity of the epidermal barrier depends on the continuous regeneration of the cornified layer. Cornification is a tightly controlled process that requires coordinated keratinocyte proliferation, detachment, migration, and cell death by terminal differentiation [25]. A striking finding in the skin of the SerpinB7^-/-^ mice was the defect in terminal keratinocyte differentiation and cornified layer formation. Previous studies have demonstrated skin barrier abnormalities in psoriasis [26, 27]. Recently, some epidermal genes have been documented as susceptibility genes for psoriasis, which are involved in the function of antimicrobial protection, innate immunity of the epidermis, and barrier function [28]. A recent study demonstrated that barrier recovery was compromised in uninvolved skin of psoriasis patients, supporting that barrier abnormality is the underlying pathogenesis of psoriasis [29]. Consistent with this, our finding that SerpinB7^-/-^ mice showed aggravated skin inflammation and epidermal hyperplasia in the IMQ-induced psoriasis-like model suggests that SerpinB7 may participate in the crosstalk between permeability barrier abnormality, cutaneous, and systemic inflammation for the development of psoriasis.

Our finding that stimulation of keratinocytes with M5 induces inflammation that aptly recapitulates the features of psoriasis as in vitro psoriatic model [30] showed that SerpinB7 expression increased with the severity of the psoriatic lesion. This is consistent with the results of IMQ-induced psoriatic lesion and psoriasis patients. A previous study identified one positive regulatory motif activator protein-1 (AP-1) binding motif (CTGATTCAC) within the –120 to –112 region of SerpinB7 [31]. IL-17 signals through IL-17RA and IL-17RC receptors to recruit the Act1 adaptor, which further recruits TRAF2, TRAF5, and TRAF6. TRAF2, and TRAF5 and stabilizes the CXCL1 mRNA, whereas TRAF6 leads to the activation of AP-1-related genes [32]. In addition, TNF-a recruits two mitogen-activated protein kinase subfamilies, the stress-activated protein kinases (SAPKs, also called JNKs) and the p38s, to transduce signals to AP-1-related genes [32]. This may be one of the mechanisms leading to the high expression of SerpinB7 in psoriatic lesions.

Despite its role in diminishing the expression of inflammatory mediators, SerpinB7 is highly expressed in psoriatic lesions. While these are seemingly contradictory findings, the phenomenon is often reported. Elafin is a skin-derived serine protease inhibitor, considered to be important in preventing human leukocyte elastase-mediated tissue damage, and might play an important role in maintaining the integrity of the human epidermis [33]. Although elafin is not detectable in normal skin, it is secreted abundantly in psoriasis [34, 35]. This is consistent with the fact that SerpinB7 is highly expressed in psoriatic lesions and inhibits the expression of inflammatory mediators.

Calcium plays important role in keratinocyte differentiation [22]. In the epidermis, calcium gradients from low levels in the proliferative basal layer to high levels in the differentiated granular layer, have been reported [22]. Dysregulated intracellular Ca^2+^ concentration ([Ca^2+^]_i_), characterized by a reduced expression of CARS, ORAI1, ORAI3, STIM1, CALB1, and TRPV6, is associated with an altered balance between keratinocyte proliferation and differentiation in the psoriatic epidermis [36, 37]. The transcriptomics and proteomics results suggested that SerpinB7 may affect [Ca^2+^]_i_ by regulating calcium ion channel relative proteins, leading to SerpinB7^-/-^ being characterized by thickening of the epidermis and severe parakeratosis. Keratinocytes are also a relevant source of chemokines and chemokine receptors [38], attract distinct immune cells into the skin during inflammatory or immune responses [39], and are significantly located in the stratum of basal and spinal [40]. Calcium flow in keratinocytes can regulate the expression of chemokines CXCL1, CXCL2, CXCL11, and antimicrobial peptides BD2, BD3, and S100A7 [40]. These results indicated that the SerpinB7 deficiency induced high expression of chemokines and antimicrobial peptides in differentiated keratinocytes, which may be due to its inhibition of keratinocyte differentiation.

Recent discoveries have highlighted that protease regulation, especially the proteolytic processing of cornification envelope conformation, plays an important role in the desquamation process of the stratum corneum and can activate and inactivate defense molecules in human epidermis [5]. Protease inhibitors such as LEKTI, elafin, SLPI, Serpins, and cystatins regulate their proteolytic activity and contribute to the integrity and protective barrier function of the skin. Changes in the proteolytic balance of the skin can result in inflammation, exhibiting typical clinical signs like redness, scaling, and itching. Pnserpin could inhibit the xylene-induced mouse ear swelling and suppress the production of proinflammatory cytokines in mouse serum and in LPS-induced RAW264.7 cells [41]. Recombinant SerpinA3 alleviated the severe inflammation in the IMQ-induced psoriasis-like mouse model [42]. SerpinB1 plays an important role in inhibiting keratinocytes proliferation [22]. and inhibits the activities of elastase, cathepsin G, and protease 3 secreted by neutrophils [43]. Moreover, PARs are transmembrane G-protein-coupled receptors, which are stimulated by proteolytic cleavage, including KLK5, KLK14, matriptase, and mast cell tryptase [44]. The release of an N-terminal pro-peptide exposes an attached ligand for the receptor at its new amino terminus, stimulating signal transduction and the activation of downstream pathways [44]. Cleavage and activation of PARs by extracellular proteases are also capable of causing [Ca^2+^]_i_ signaling [45]. PAR-1, PAR-2, and PAR-4 agonist peptides significantly and abruptly increased [Ca^2+^]_I_ [46]. This suggests that protease inhibitors can protect the integrity of the skin barrier and inhibit inflammatory responses. However, further investigations are needed to determine whether SerpinB7 affects keratinocytes by inhibiting protease activity.

In summary, this study found that SerpinB7, a gene specifically expressed by keratinocytes, was associated with the occurrence and development of psoriasis, through transcriptomic screening and identification. It was found that SerpinB7 was positively correlated with the severity of psoriasis. SerpinB7 deficiency affects the skin barrier function and the expression of inflammatory mediators in mice, and exacerbates psoriasis-like lesions. Through transcriptomics and proteomics analyses, it was found that the loss of SerpinB7 affects the differentiation process of keratinocytes and the expression of calcium signaling pathway-related genes, and experiments have verified that the loss of SerpinB7 may inhibit keratinocytes by inhibiting the calcium ion concentration in keratinocytes, thereby inhibiting keratinocyte differentiate and promoting the expression of keratinocytes and inflammatory mediators, affecting the occurrence and development of psoriasis. This study explored the relationship between SerpinB7 and psoriasis, analyzes the mechanism of regulating psoriasis keratinocytes, and provides new ideas for further understanding of the skin homeostasis regulatory network and the occurrence and development of psoriasis. It also provides a possible experimental basis and theoretical basis, implying a new keratinocytes-specific candidate for therapeutic targets in psoriasis.

## Materials and methods

### Patients

Skin samples were obtained from 13 psoriasis patients and 15 healthy donors with a 2-mm punch biopsy. None of the participants had received systemic therapy, including investigational agents at least 4 weeks before entering the study. Patients with preexisting autoimmune disease, immunologic deficiency diseases, or tumors were excluded. All these samples were used for RNA isolation or paraffin section. This study was authorized and approved by the Ethics Committee of Sichuan University (No. [2020]234).

### RT-PCR

Total RNA was extracted with TRIzol (Thermo Fisher Scientific) according to the manufacturer’s protocol, followed by quality control using capillary electrophoresis (NanoDrop 2000; Thermo Fisher Scientific). RNA (1 ug) was reversed transcribed using a PrimeScript RT reagent kit with gDNA Eraser (Takara Bio; RR047A). RT-PCR reactions were carried out with gene-specific primers (Qing Ke Bio) mixed with SYBR Green PCR Master Mix (Takara Bio; RR820), according to the manufacturer’s protocol. Samples were run in triplicates in a LightCycler96 PCR system (Roche). mRNA expression was normalized using ACTB as a reference. Analysis was performed according to the ΔΔCt method (see Supplementary Table for primer sequences).

### Immunoblot

Total proteins were extracted in RIPA buffer (Sigma-Aldrich) supplemented with anti-protease (Roche). Protein concentrations were determined using the Micro BCA Protein Assay Kit (Thermo Fisher Scientific). Proteins (50 ug) were separated by electrophoresis in SDS-PAGE gels and transferred onto polyvinylidene fluoride membranes (Merck Millipore, IPVH0010). After membrane probing with primary antibody, horseradish peroxidase-conjugated secondary antibodies (Pierce) were used for signal detection in immunoblot ELC (WBULS0500; Merck Millipore). The primary antibodies used were: SerpinB7 (Sigma-Aldrich, HPA024200, Biorbyt, orb11036).

### Histology, immunohistochemistry, immunofluorescence staining

Human and mice skin tissues were fixed with 4% paraformaldehyde in PBS, embedded in paraffin, sectioned, and stained with H&E for histopathologic examination. Immunohistochemistry was performed on formalin-fixed, paraffin-embedded skin tissues. The slices were probed overnight with SerpinB7 (Sigma-Aldrich, HPA024200), mKi67 (Abcam, ab16667). For Immunofluorescence, skin frozen sections were cryosectioned, blocked, and then stained with anti-Filaggrin (Biolegend, 905804), anti-KRT10 (Abcam, ab76318), anti-Loricrin (Biolegend, 905101). Images were captured using an Olympus BX600 microscope (Olympus Corporation, Tokyo, Japan) and SPOT Flex camera (Olympus Corporation, Tokyo, Japan) and were analyzed with ImagePro Plus (version 6.0, Media Cybernetics) software. The epithelial thickness and infiltrating cells were calculated in independent regions as described previously [47].

### Genome-wide transcriptome profiling by RNA-Seq

NHEK were cultured in vitro for 2 days, then treated with 1.6 mM Ca^2+^ for 24 h. Total RNA was isolated with Trizol (Invitrogen), and subjected to RNA-seq analysis. RNA sequencing was performed by Novogene (Beijing, China) information technology Inc. The raw reads were aligned to the mm10 reference genome (build mm10) by using HISAT2 software. The mapping rate was more than 90% overall across all the samples. StringTie was used to quantify the gene expression counts. Differential expression analysis was performed on the count data using R package DESeq2. Benjamini–Hochberg step-up method to control false discovery rate. Significant genes are defined by a Benjamini and Hochberg corrected *P* value of cut-off of 0.05 and fold-change of at least 2.

### Dye and CEs

For toluidine blue staining, newborn mice were sacrificed and dehydrated by sequential incubation in 25, 50, 75, and 100% methanol. After rehydration in PBS, they were incubated for 10 min in 0.01% toluidine blue and detained with PBS.

CEs were isolated as previously described [48]. CEs were prepared from a defined area of newborn dorsal mouse skin (25 mm^2^) by boiling in a buffer consisting of 20 mM Tris-HCl, pH7.5, 5 mM EDTA, 10 mM DTT, and 0.2% SDS. For sonication, CEs were suspended in a 2% SDS solution.

### Weighted gene co-expression network analysis

The weighted gene co-expression network analysis package in R was used for step-by-step network construction and module detection of skin tissue-specific genes analyzed from the GTEx database [49]. The soft thresholding power *β* = 4 was selected based on the criterion of scale-free topology. Modules were identified with a minimum module size of 15 mRNAs. GO analyses of the three largest modules (blue, turquoise, and brown) were performed, and an interactive network of the module involving SerpinB7 was visualized using Cytoscape.

### Animals

SerpinB7^-/-^ mice were provided by Cyagen Biosciences (Guangzhou, China). Briefly, to create a SerpinB7 knockout mouse model (C57BL/6) by CRISPR/Cas-mediated genome engineering, SerpinB7 exon 5 was selected as the target site. Cas9 and gRNA (gRNA1: GTATAATTAAAGCACCGATGTGG, gRNA2: GGATAGCGAGTCAACATCTCTGG) were co-injected into fertilized eggs for KO mouse production, the pups were genotyped by PCR followed by sequencing analysis. Primers used as follows: F1: 5’-GGAGTGCAATAGTAAACAAAGC-3’, R1: 5’-TGTACATACCGTGGGAGACC-3’, R2: 5’-TGCCTCCAAAAAGTGAAAAC-3’. The method of randomization was the random number table. The mice were raised in standard housing conditions (22 ± 1 °C; light/dark cycle of 12/12 h), with water and food available ad libitum. All experiments were performed in accordance with the US National Institutes of Health Guide for the Care and Use of Laboratory Animals (NIH Publication No. 8023) and its 1978 revision. All of the experiments comply with the animal study protocol approved by the Laboratory Animal Welfare and Ethics Committee of Sichuan University.

### Cell lines

The keratinocyte cell line HaCaT was obtained from the China Center for Type Culture Collection (0106). Human embryonic kidney cells (HEK293T) were purchased from the ATCC (CRL-1573), and the HaCaT or HEK293A cells were cultured in Dulbecco’s modified Eagle’s medium (DMEM; Thermo Fisher Scientific, C11995500BT) supplemented with 10% (v:v) fetal bovine serum (FBS; Thermo Fisher Scientific, 10099141), 100 U/mL penicillin G and 0.1 mg/mL streptomycin sulfate (Thermo Fisher Scientific, 15140122). The cell culture conditions were 37 °C and 5% CO_2_. All cells were found to be free from mycoplasma contamination.

### Preparation of primary keratinocytes

Primary NHEKs were isolated from the newborn circumcision foreskin with donors’ agreement and from the adult abdomen with the donors’ consent using an enzyme digestion method, as described in the literature [50]. Primary normal MKs were isolated from the neonatal and adult mouse skin as previously described [51]. Briefly, the skin samples were cut into small pieces and incubated in 2.4 U/mL dispase II (CELLnTEC, CnT-DNP-10) solution overnight at 4 °C to allow separation of the epidermis from the dermis. The epidermis was used for the isolation of primary keratinocytes using 0.25% trypsin-EDTA (Life Technologies, 25200072). The primary keratinocytes were cultured in the CnT-PR medium (CELLnTEC, CnT-PR) supplemented with IsoBoost (CELLnTEC, CnT-ISO-50) for at least the first 3 days post seeding and then switched to standard CnT-PR medium. For all the experiments, the primary keratinocytes were used after two passages to ensure the absence of contaminating cells.

### Lentiviral gene silencing and overexpression in vitro

Lentiviral constructs with shRNAs were directed against murine and homo *SerpinB7* in the pLKO.1 vector and overexpressed homo SerpinB7 in pCDH-CMV vector. Lentivirus was prepared by transient transfection of HEK293T cells with transfer vectors along with second-generation packaging constructs (pMD2.G and psPAX2). The viral titers were determined with qPCR. HaCaT, primary keratinocytes were transfected with uncentrated virus supernatant overnight in the presence of polybrene (5 mg/mL) and selected in puromycin (0.5 mg/mL).

### Induction of the in vitro psoriatic model

NHEK cells or HaCaT cells were stimulated with 10 ng/mL recombinant IL17A (Prospec Protein Specialists, CYT-250), OSM (Prospec Protein Specialists, CYT-231), TNF-α (Prospec Protein Specialists, CYT-223), IL22 (Prospec Protein Specialists, CYT-328), and IL-1α (Prospec Protein Specialists, CYT-253) alone or in combination (named M5 combination) in DMEM supplemented with 2% (v:v) FBS, to recapitulates numerous features of psoriasis [30, 52].

### Induction of the in vivo psoriatic model

Ten-week-old mice were given 27.5 ug of IMQ in shaved back daily for 5 days as previously described [47]. Briefly, mice were shaved on the back one day before induction, and treated with Aldara cream (Sichuan MingXin Pharmaceutical Co., LTD., H20030129) containing 5% IMQ (55 mg) once daily for 5 days. To measure the severity of inflammation on the back, a scoring system considering skin thickness, scaling, and erythema, (not taking into account the area which was determined by the experimenter) similar to the human PASI score was used (cumulative IMQ score) [20].

IL-23-dependent psoriatic inflammation models were established as previously described [53]. Briefly, 10-week-old mice were injected intradermally with recombination IL-23 (Sino, CT028-M08H) in two locations on either side of the shaved back for a total of 1 ug protein per mouse using a 29.5-gauge needle. Injections were performed daily for 5 days.

### Keratinocyte differentiation

Calcium-induced keratinocyte differentiation methods were performed as previously described [54]. Briefly, to promote terminal differentiation, a calcium switch was performed by adjusting the calcium level to the final concentration of 1.6 mM CaCl_2_ (Sigma, 449709) for a period of 24–72 h before harvest, and changed the medium every 24 h. Recombination SerpinB7(abnova, H00008710) were added 30 min before Ca^2+^ stimulation.

### Measurement of intracellular Ca^2+^ and mitochondrial Ca^2+^ level

The intracellular Ca^2+^ and mitochondrial Ca^2+^ levels of indicated cells were determined according to the manufacturer’s instruction using Fluo-4-AM (Thermo Fisher Scientific) and Rhod2-AM (Thermo Fisher Scientific), respectively. In brief, cells were stained with 1 µM Fluo-4-AM, 1 µM Rhod2-AM for 30 min. After staining, each fluorescent intensity was determined using a flow cytometer and immunofluorescence staining.

### Statistical analysis

All statistical analysis was performed with GraphPad Prism 8 software (GraphPad Software Company, version 8.0.). Student’s *t*-test was used for comparing two groups, Spearman’s rank correlation test was used to analyze the relationship between two quantitative variables, and a one-way analysis of variance with Turkey’s post hoc test was utilized for the comparisons of multiple groups. *P* < 0.05 was considered statistically significant.

## Supplementary information

Supplementary figure legends

Supplementary table legends

Supplementary Figure 1

Supplementary Figure 2

Supplementary Figure 3

Supplementary Figure 4

Supplementary Figure 5

Supplementary Figure 6

Supplementary Figure 7

Supplementary Figure 8

Table 1

Table 2

Original Data File

## Data Availability

All datasets generated and analyzed during this study are included in this published article and its Supplementary information files. Additional data are available from the corresponding author on reasonable request.
