# Supplementary material for: SerpinB7 deficiency contributes to development of psoriasis via calcium-mediated keratinocyte differentiation dysfunction
Source: Cell Death Dis. 2022 Jul 21;13(7):635. doi: 10.1038/s41419-022-05045-8 (PMC9304369; doi:10.1038/s41419-022-05045-8)
Supplement: Supplementary file 1 — Supplementary figure legends [file 41419_2022_5045_MOESM1_ESM.docx]

**Supplementary Figure 1**

Expression analysis of SerpinB7 in clinical psoriasis samples using public GEO data (GDS4602, GDS4600, GDS5420).

**Supplementary Figure 2**

HaCaT were stimulated with M5 or respectively for 0 to 48h, n=3/group. (A-B) The RNA levels of SerpinB7 were assessed by qPCR. (C) Representative immunoblot of SerpinB7 at 24 and 48h.

**Supplementary Figure 3**

Creating SerpinB7^-/-^ mice by CRISPR/Cas9-mediated genome engineering. (A) Eight exons have been identified. Exon 5 was Selected as target sites. (B) Screening of SerpinB7^-/-^ mice by PCR, the PCR products for SerpinB7^+/+^: only 704bp, Heterozygous: 911bp and 704bp; KO: only 911bp. (C)The skin tissue were isolated from SerpinB7^-/-^, SerpinB7^+/-^ and SerpinB7^+/+^ mice, qPCR analysis of SerpinB7 gene transcription, each circle represents one mouse. (D) Immunoblot analysis of SerpinB7 protein. (E) Macroscopic appearance of SerpinB7^-/-^ mice and wildtype control. (E) Body weight over time.

**Supplementary Figure 4**

SerpinB7 deficiency attenuated rhIL-23 induced psoriatic inflammatory model. (A) Representative H&E staining section (20 x) from the skin tissues of SerpinB7^+/+^ and Serpinb7^-/-^ mice; (B) The mRNA levels of keratinocyte differentiation markerswere measured by RT-PCR; (C) The mRNA levels of cytokines, chemokines and antimicrobial peptides markers were measured by RT-PCR.

**Supplementary Figure 5**

SerpinB7 inhibit M5 stimulated psoriatic invitro model chemokines and cytokines expression. (A) HaCaT cells were infected with SerpinB7 shRNA Lentivirals. The mRNA level of SerpinB7 was measured by RT-PCR. (B) The mRNA levels of chemokines (Ccl20, Ccl27, Cxcl1 and Cxcl2) and antimicrobial peptides (LL37 and S100a8) were measured by RT-PCR. (C) HaCaT cells were infected with SerpinB7 overexpression Lentivirals. The mRNA level of SerpinB7 was measured by RT-PCR. (D) The mRNA levels of chemokines (Ccl20, Ccl27, Cxcl1, Cxcl2 and Cxcl8) and antimicrobial peptides (BD2, LL37, S100a7, S100a8, S100a9 and S100a12) were measured by RT-PCR.

**Supplementary Figure 6**

(A-B) The top 10 most relevant upregulated and downregulated DEGs Kyoto Encyclopedia of Genes and Genomes (KEGG) pathways are sorted according to the adjusted *p*-value. (C-D) STRING protein-protein interaction (PPI) analyses in upregulated and downregulated DEGs.

**Supplementary Figure 7**

(A) SerpinB7 gene expression in tissues from GTEx RNA-seq of 607 samples across 51 tissues. (B) SerpinB7 RPKMs in descending order in skin tissues of GTEx database. (C) Heat map showing hierarchical cluster analysis of mRNAs related to keratinocyte differentiation in two groups (D) Gene ontology terms enriched in downregulated genes. (E-F) SerpinB7 mRNA and protein expression in Ca2+ induction NHEKs. (G) Loricrin, Filaggrin, Krt10 and Krt5 mRNA levels serve as internal control.

**Supplementary Figure 8**

(A) Ca^2+^ concentration in cytosol ([Ca^2+^]_c_) of lentiviral and exogenous recombination protein overexpressed SerpinB7 NHEKs homeostasis and treated for 24h with 1.6mM Ca^2+^ were measured by flow cytometry using Flou4-AM; Keratinocyte differentiation markers (B) and calcium channel related-markers (C) were measured in oeSerpinB7 and shSerpinB7 NHEKs after 1.6 mM Ca^2+^ and exogenous recombination SerpinB7 treated.
