# Supplementary material for: SerpinB7 deficiency contributes to development of psoriasis via calcium-mediated keratinocyte differentiation dysfunction
Source: Cell Death Dis. 2022 Jul 21;13(7):635. doi: 10.1038/s41419-022-05045-8 (PMC9304369; doi:10.1038/s41419-022-05045-8)
Supplement: Supplementary file 2 — Supplementary table legends [file 41419_2022_5045_MOESM2_ESM.docx]

**Supplementary Table 1**

Differentiated *SerpinB7* shRNA or scramble shRNA HaCaT Cell line RNA-seq complete gene list.

**Supplementary Table 2**

Differentiated SerpinB7 knockout NHEK cells proteomic complete gene list and GO categories
