# Supplementary figures and images for: SerpinB7 deficiency contributes to development of psoriasis via calcium-mediated keratinocyte differentiation dysfunction
Source: Cell Death Dis. 2022 Jul 21;13(7):635. doi: 10.1038/s41419-022-05045-8 (PMC9304369; doi:10.1038/s41419-022-05045-8)

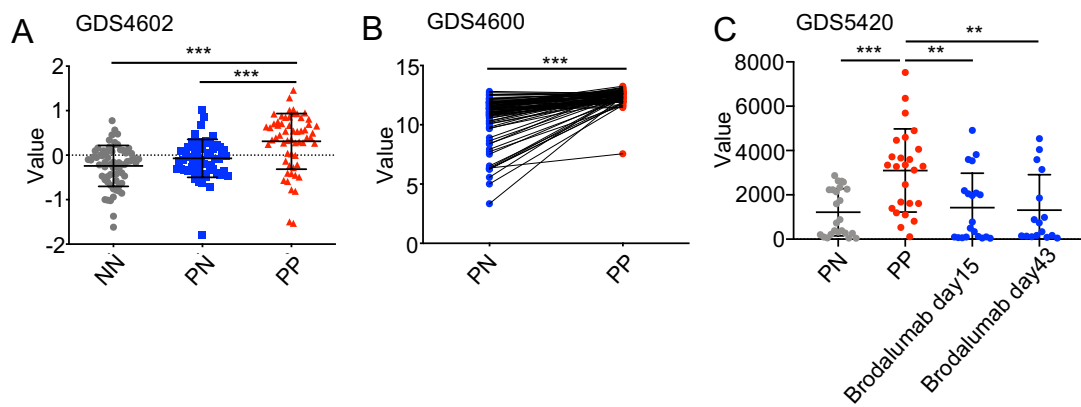

Supplement: Supplementary file 3 — Supplementary Figure 1 [file 41419_2022_5045_MOESM3_ESM.pdf]

**sFigure 2**

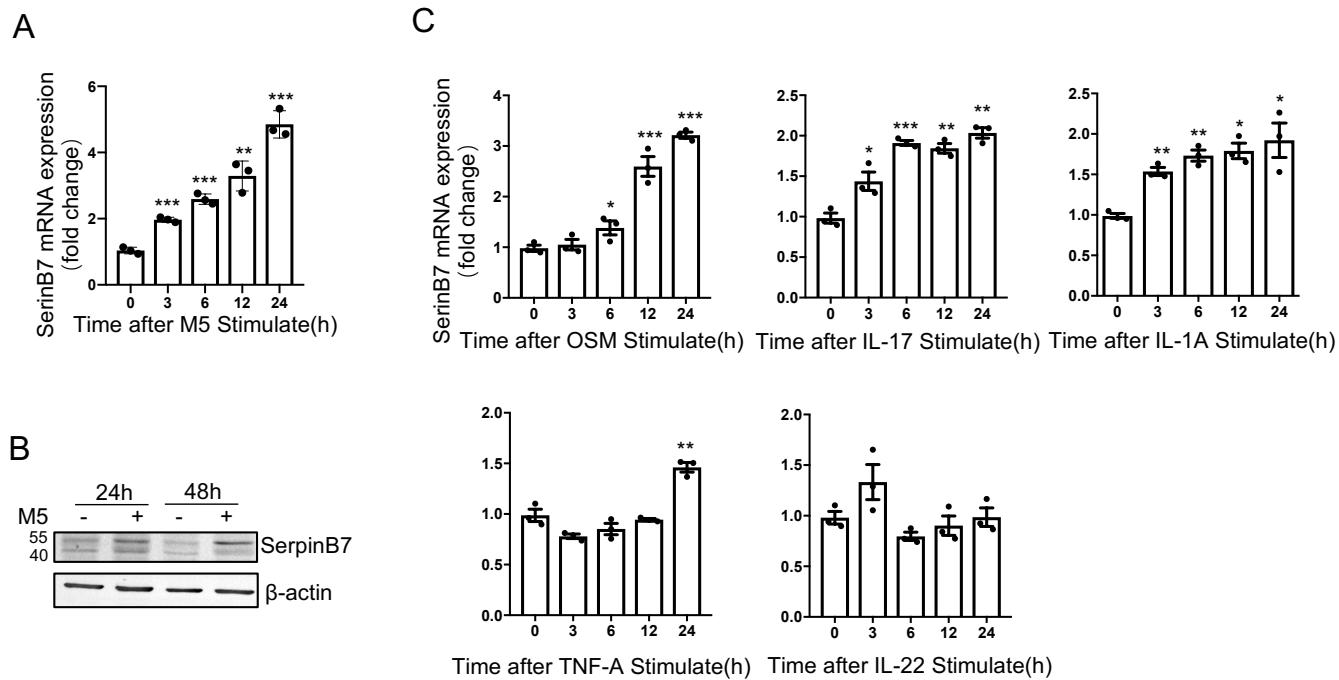

Supplement: Supplementary file 4 — Supplementary Figure 2 [file 41419_2022_5045_MOESM4_ESM.pdf]

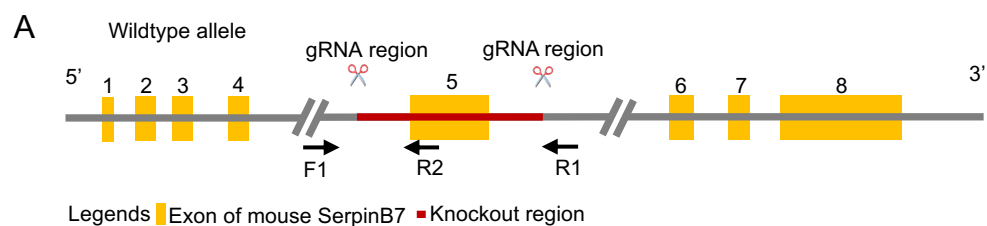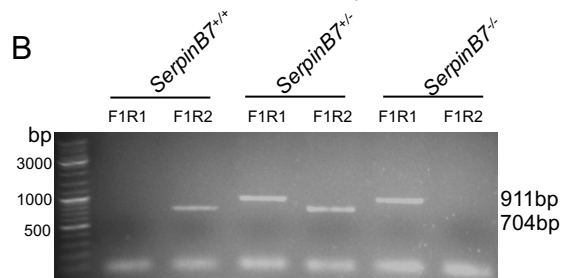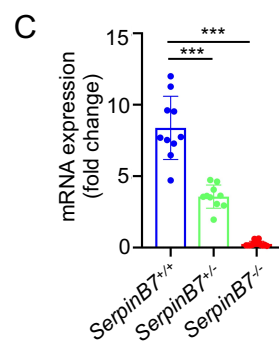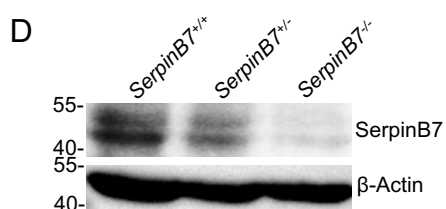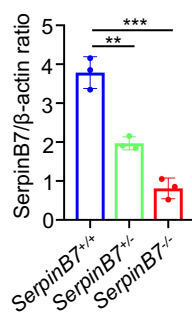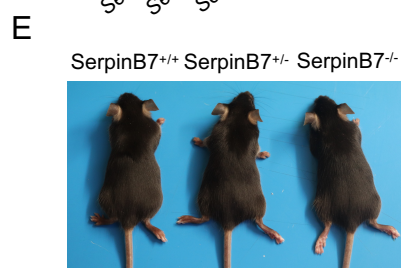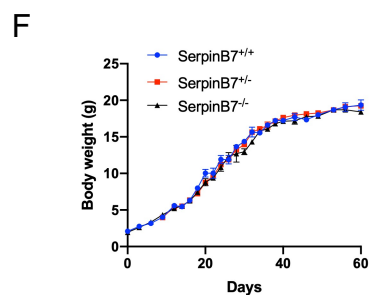

Supplement: Supplementary file 5 — Supplementary Figure 3 [file 41419_2022_5045_MOESM5_ESM.pdf]

**A**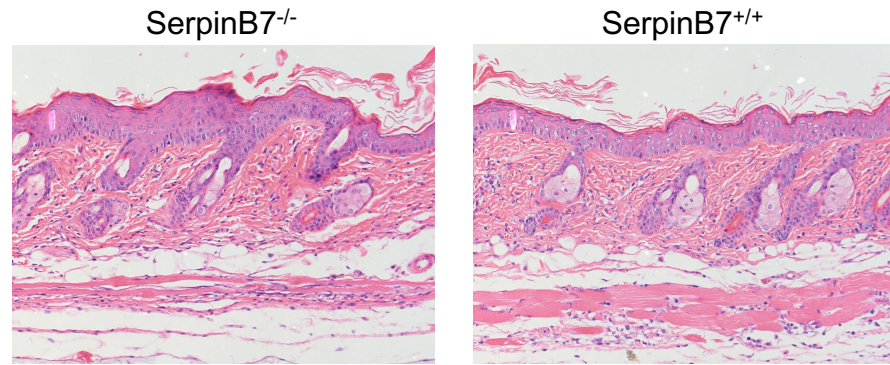**B**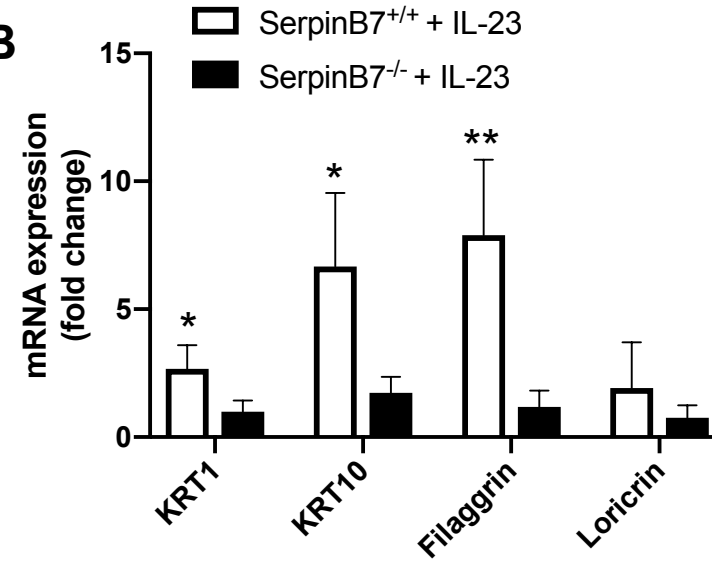**C**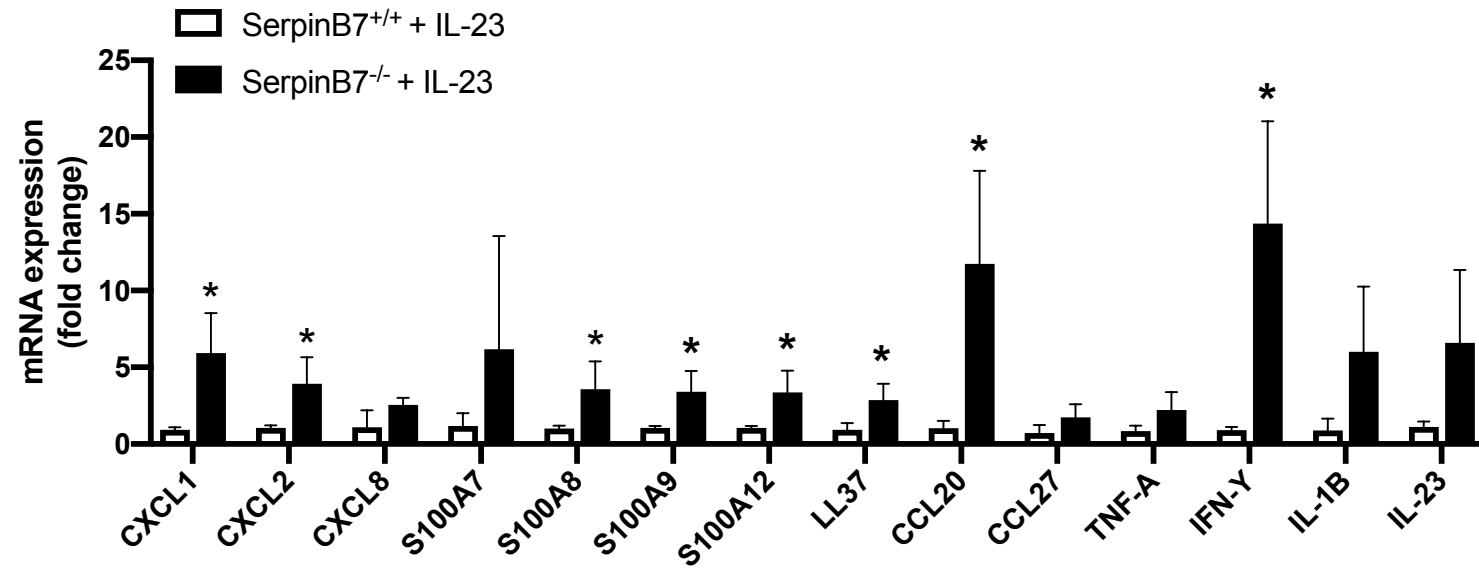

Supplement: Supplementary file 6 — Supplementary Figure 4 [file 41419_2022_5045_MOESM6_ESM.pdf]

A

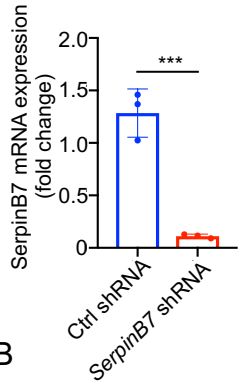

C

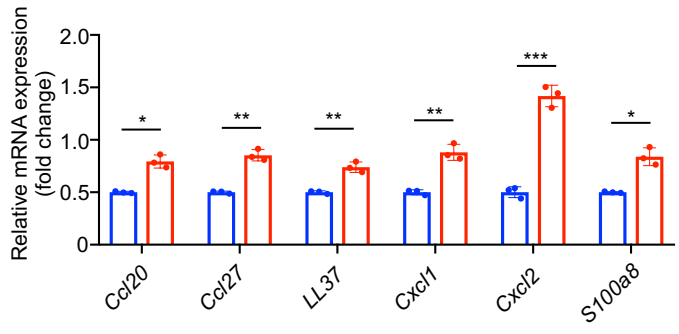

B

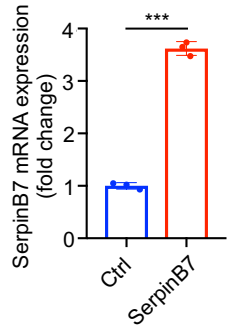

D

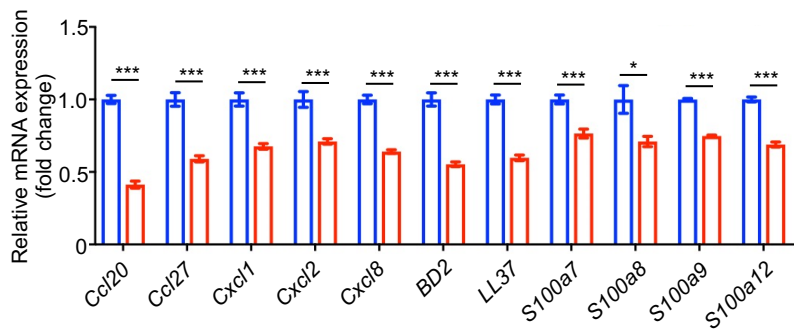

Supplement: Supplementary file 7 — Supplementary Figure 5 [file 41419_2022_5045_MOESM7_ESM.pdf]

A

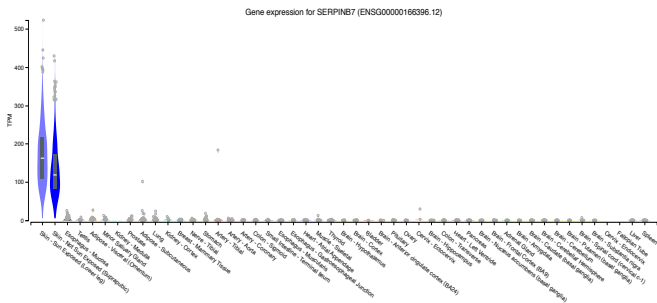

B

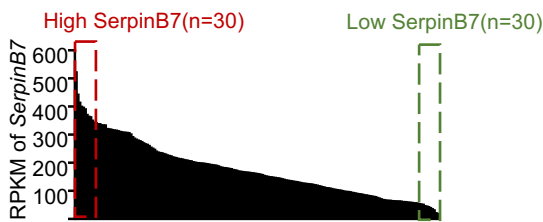

C

Keratinocyte differentiation heatmap

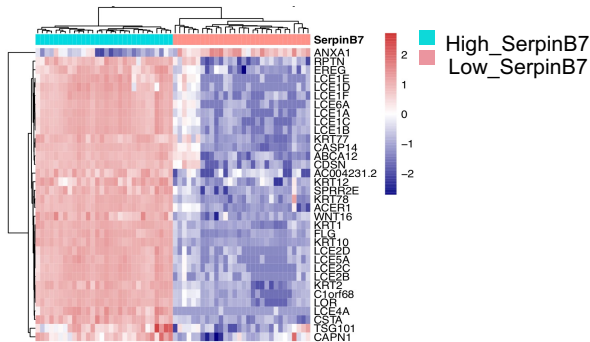

D

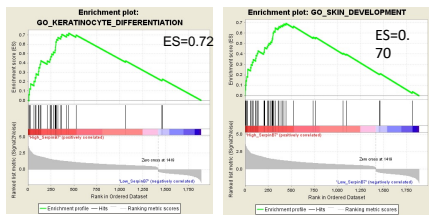

E

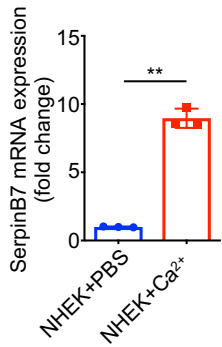

F

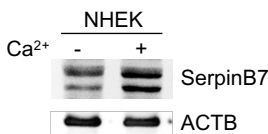

G

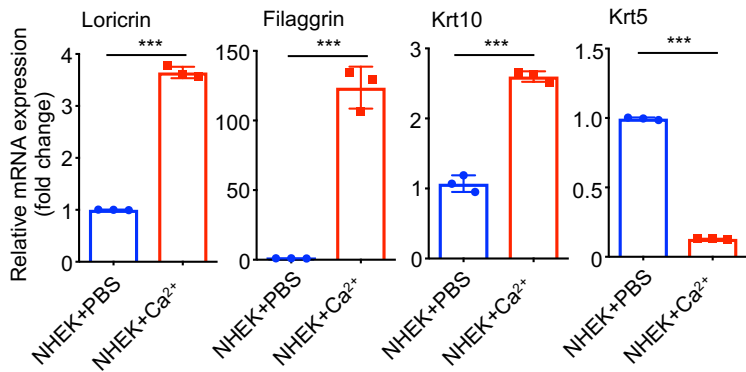

Supplement: Supplementary file 8 — Supplementary Figure 6 [file 41419_2022_5045_MOESM8_ESM.pdf]

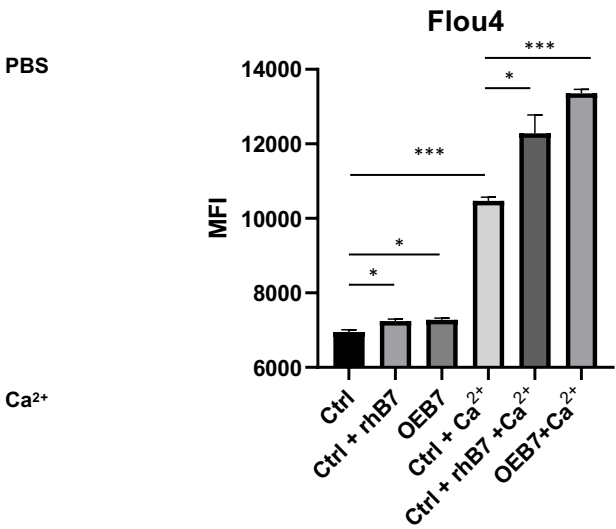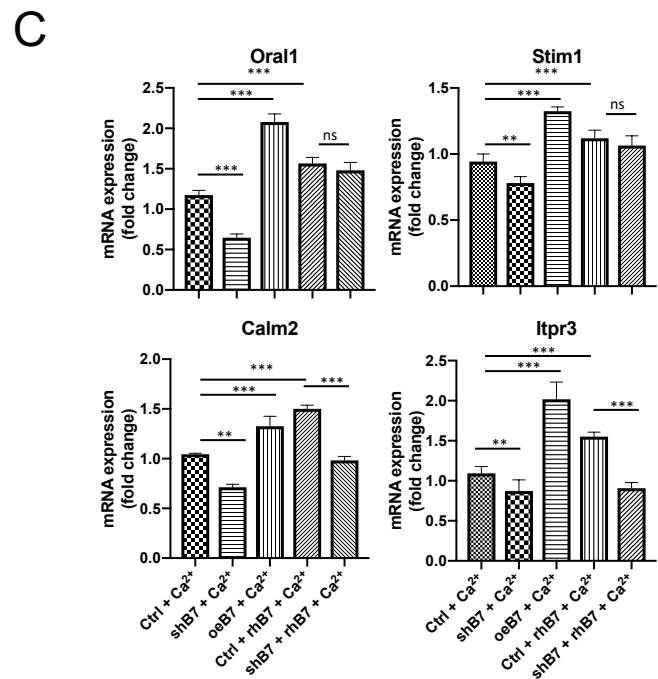

Supplement: Supplementary file 10 — Supplementary Figure 8 [file 41419_2022_5045_MOESM10_ESM.pdf]

WB原始数据


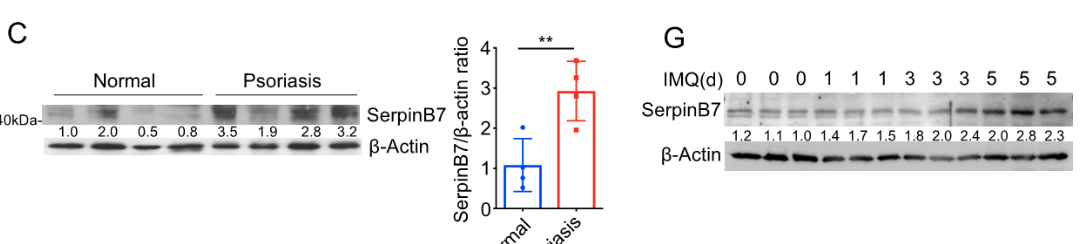


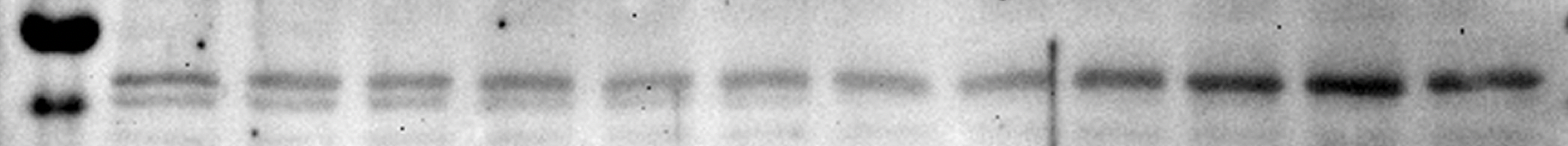


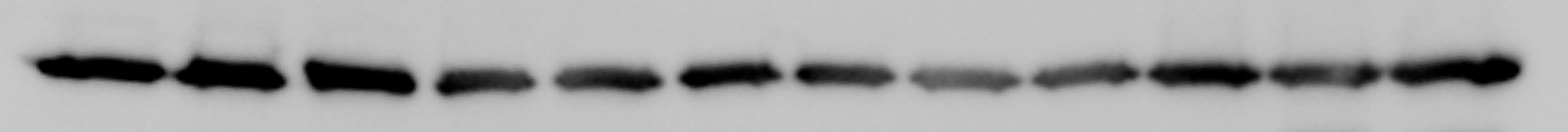


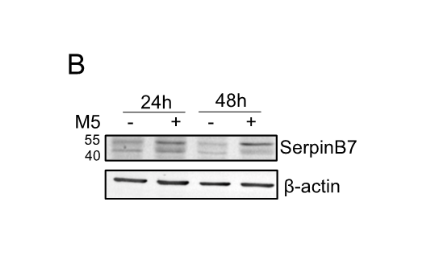


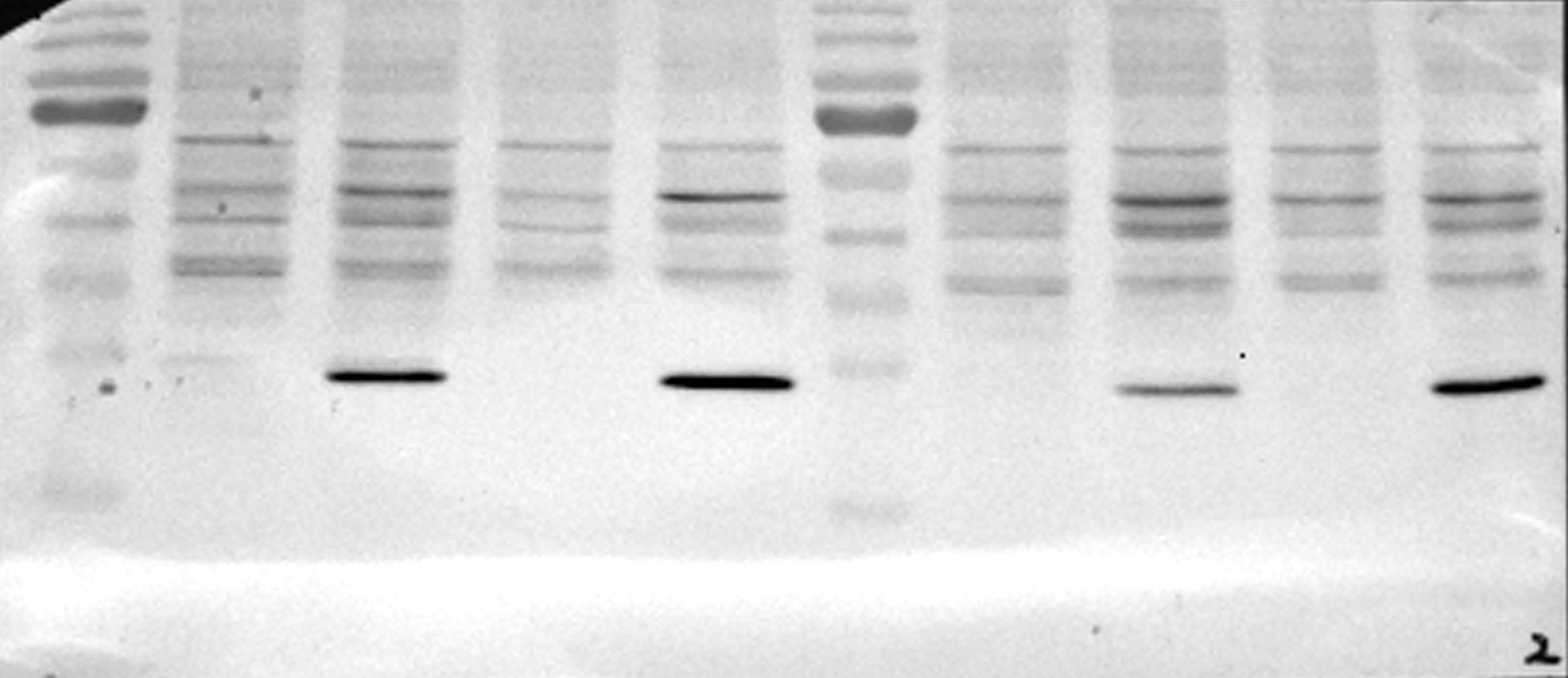


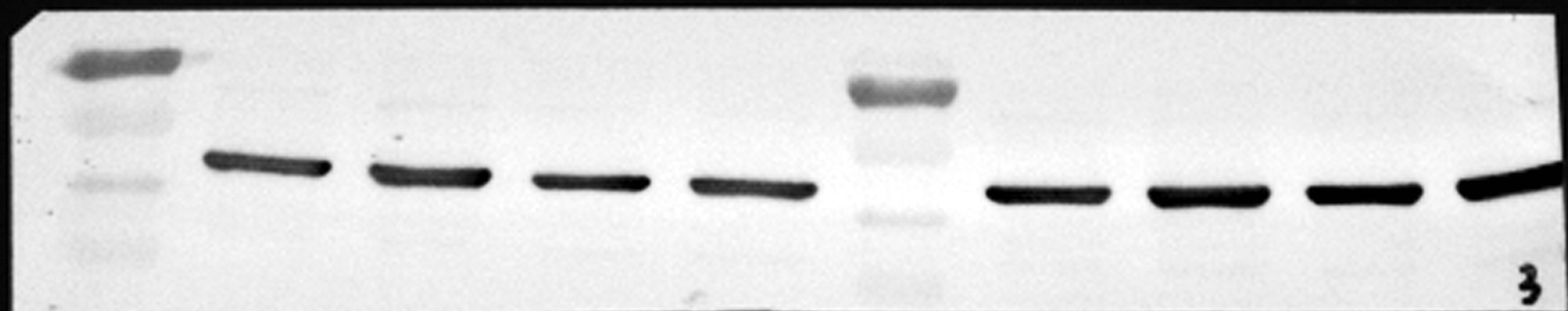


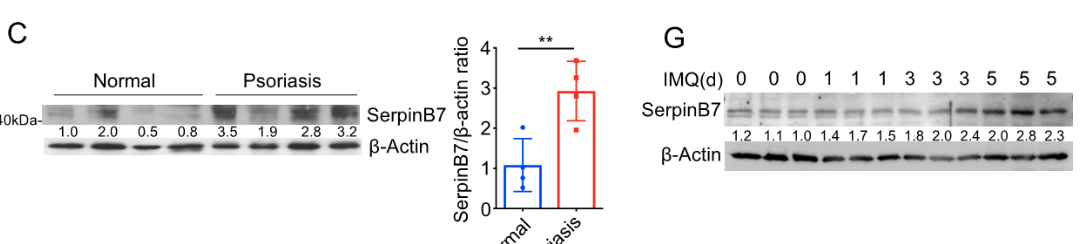


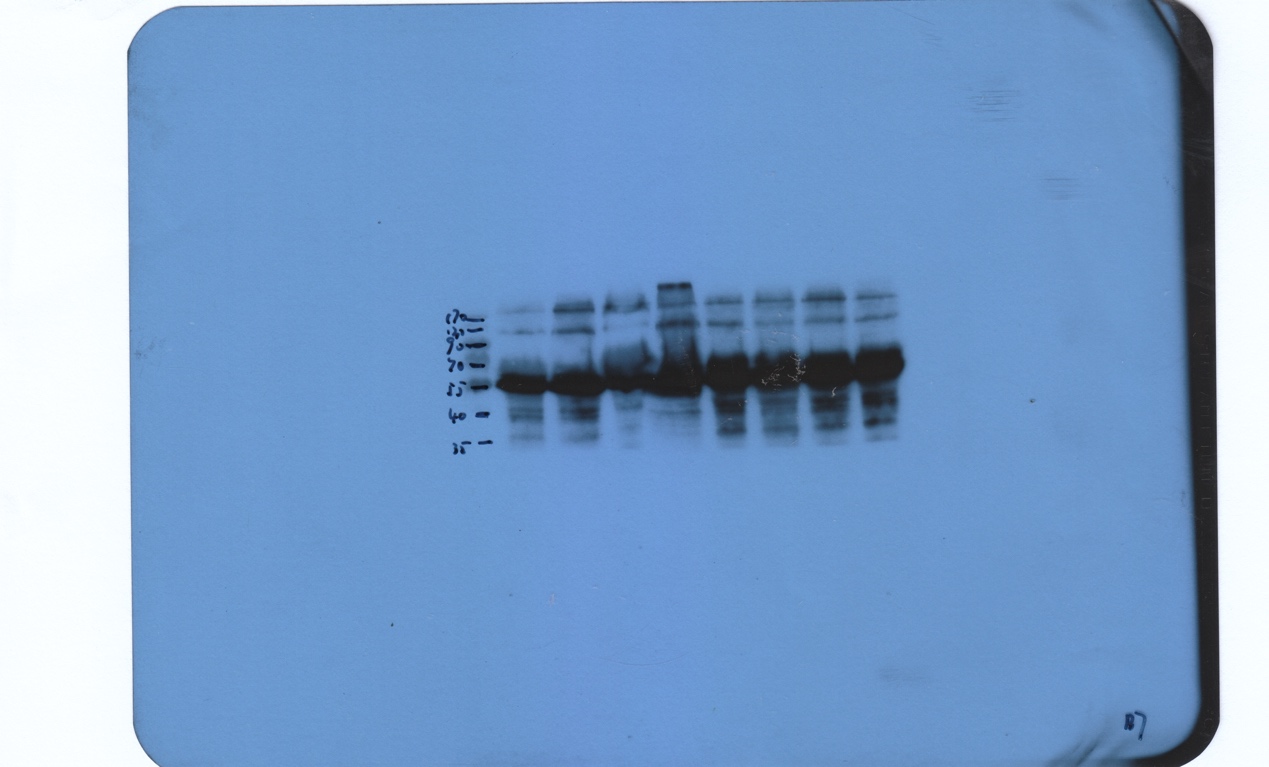


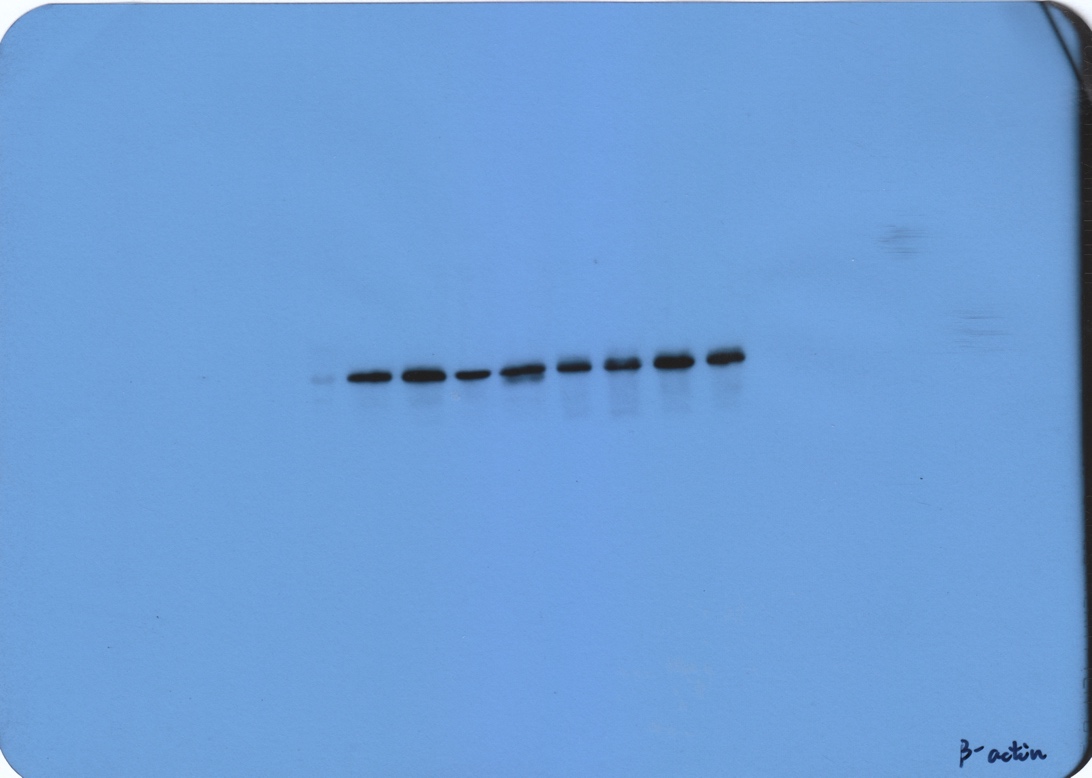


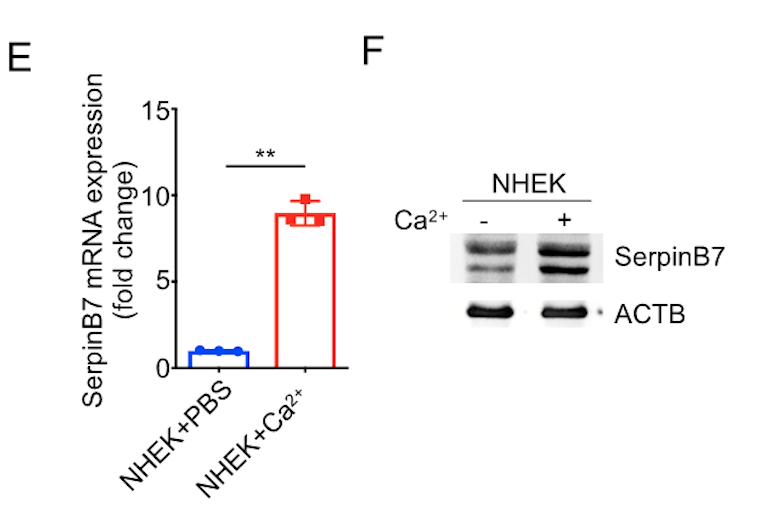


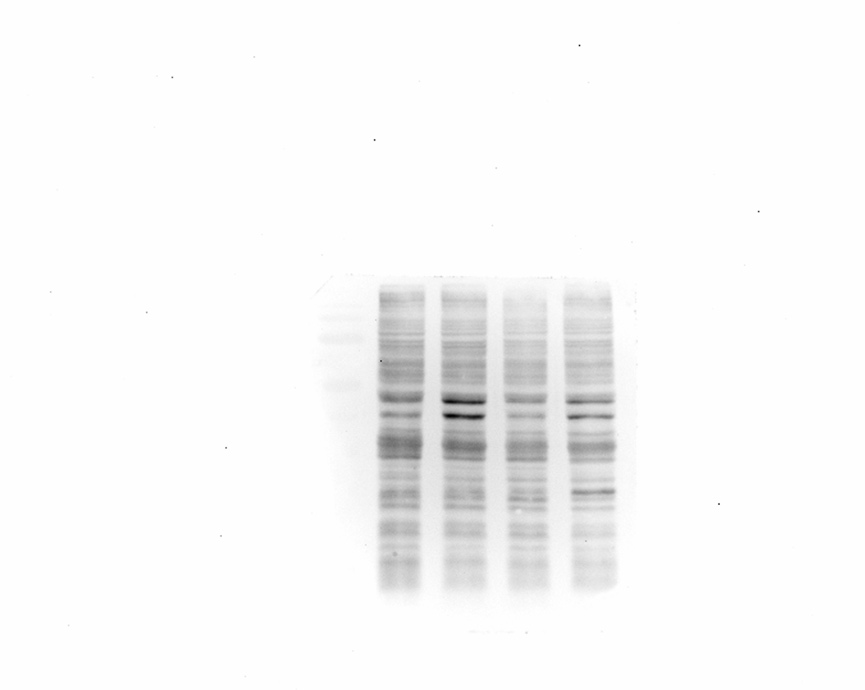


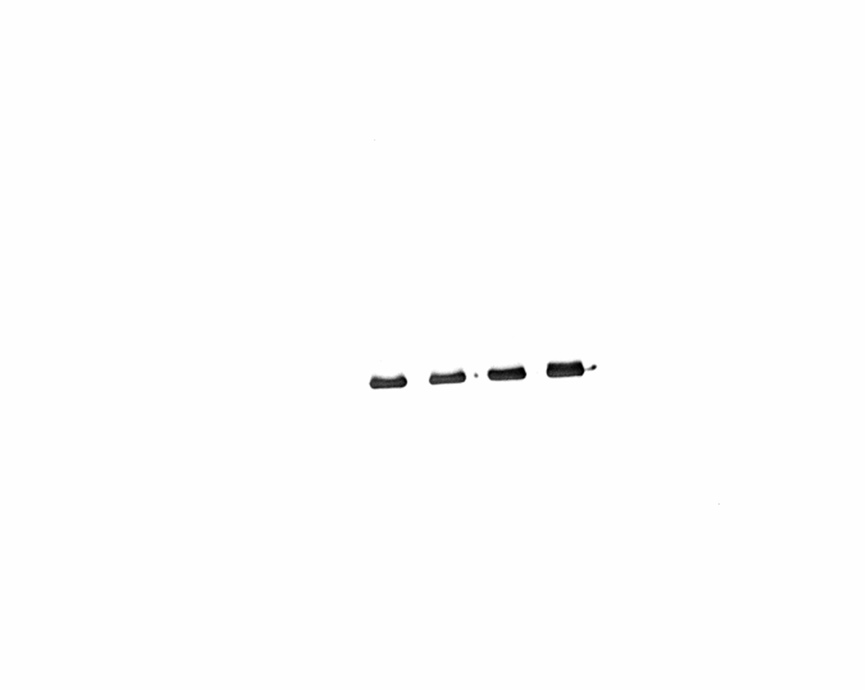

Supplement: Supplementary file 13 — Original Data File [file 41419_2022_5045_MOESM13_ESM.docx]
