# Supplementary material for: SerpinB7 deficiency contributes to development of psoriasis via calcium-mediated keratinocyte differentiation dysfunction
Source: Cell Death Dis. 2022 Jul 21;13(7):635. doi: 10.1038/s41419-022-05045-8 (PMC9304369; doi:10.1038/s41419-022-05045-8)
Supplement: Supplementary file 9 — Supplementary Figure 7 [file 41419_2022_5045_MOESM9_ESM.pdf]

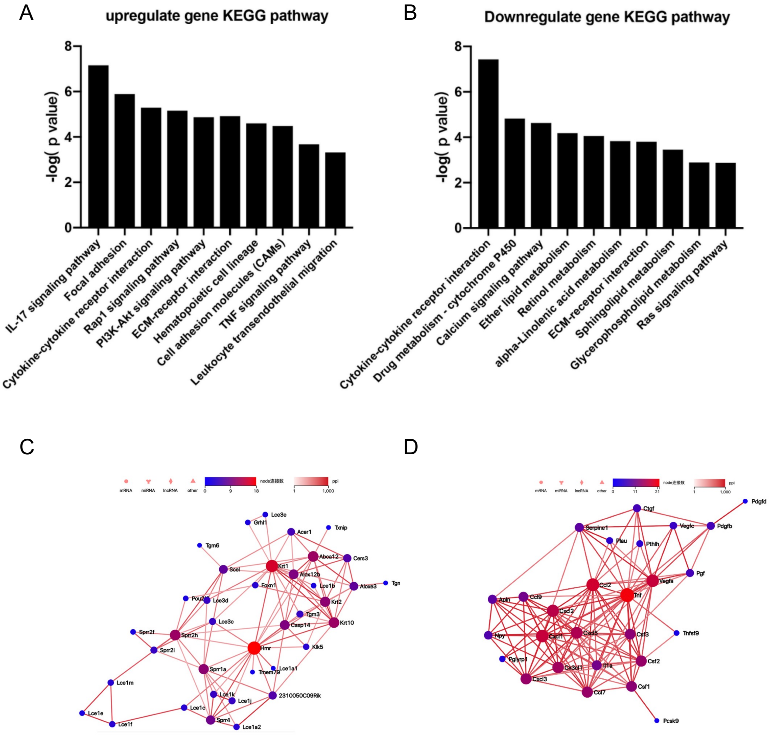
A
upregulate gene KEGG pathway
-log(p value)
0
2
4
6
8
IL-17 signaling pathway
Focal adhesion
Cytokine-cytokine receptor interaction
Rap1 signaling pathway
PI3K-Akt signaling pathway
ECM-receptor interaction
Hematopoietic cell lineage
Cell adhesion molecules (CAMs)
TNF signaling pathway
Leukocyte transendothelial migration
B
Downregulate gene KEGG pathway
-log(p value)
0
2
4
6
8
Cytokine-cytokine receptor interaction
Drug metabolism - cytochrome P450
Calcium signaling pathway
Ether lipid metabolism
Retinol metabolism
alpha-Linolenic acid metabolism
ECM-receptor interaction
Sphingolipid metabolism
Glycerophospholipid metabolism
Ras signaling pathway
C
D
